# S100A8-mediated metabolic adaptation controls HIV-1 persistence in macrophages in vivo

**DOI:** 10.1038/s41467-022-33401-x

**Published:** 2022-10-11

**Authors:** Fernando Real, Aiwei Zhu, Boxin Huang, Ania Belmellat, Alexis Sennepin, Thomas Vogl, Céline Ransy, Marc Revol, Riccardo Arrigucci, Anne Lombès, Johannes Roth, Maria Laura Gennaro, Frédéric Bouillaud, Sarra Cristofari, Morgane Bomsel

**Affiliations:** 1 Laboratory of Mucosal Entry of HIV and Mucosal Immunity, Institut Cochin, Université Paris Cité, 75014 Paris, France; 2 CNRS, UMR8104, 75014 Paris, France; 3 Inserm, U1016, Institut Cochin, 75014 Paris, France; 4 Institute of Immunology and Interdisciplinary Center for Clinical Research, University of Münster, Münster, Germany; 5 Plastic, Reconstructive and Aesthetic Surgery Department, Saint Louis Hospital, Paris, France; 6 Public Health Research Institute, New Jersey Medical School, Rutgers, The State University of New Jersey, Newark, NJ USA

**Keywords:** HIV infections, Mucosal immunology, Mechanisms of disease

## Abstract

HIV-1 eradication is hindered by viral persistence in cell reservoirs, established not only in circulatory CD4^+^T-cells but also in tissue-resident macrophages. The nature of macrophage reservoirs and mechanisms of persistence despite combined anti-retroviral therapy (cART) remain unclear. Using genital mucosa from cART-suppressed HIV-1-infected individuals, we evaluated the implication of macrophage immunometabolic pathways in HIV-1 persistence. We demonstrate that ex vivo, macrophage tissue reservoirs contain transcriptionally active HIV-1 and viral particles accumulated in virus-containing compartments, and harbor an inflammatory IL-1R^+^S100A8^+^MMP7^+^M4-phenotype prone to glycolysis. Reactivation of infectious virus production and release from these reservoirs in vitro are induced by the alarmin S100A8, an endogenous factor produced by M4-macrophages and implicated in “sterile” inflammation. This process metabolically depends on glycolysis. Altogether, inflammatory M4-macrophages form a major tissue reservoir of replication-competent HIV-1, which reactivate viral production upon autocrine/paracrine S100A8-mediated glycolytic stimulation. This HIV-1 persistence pathway needs to be targeted in future HIV eradication strategies.

## Abstract (web summary)

HIV-1 eradication is hindered by viral persistence in different cell reservoirs, including circulatory CD4+ T-cells and tissue-resident macrophages. Here, by analyzing male genital mucosa from cART-suppressed HIV1-infected individuals, Real et al. show that M4 macrophages represent the major macrophage HIV-1 reservoir in this tissue. These macrophages have an inflammatory IL1R^+^S100A8^+^MMP7^+^M4-phenotype, and contain transcriptionally active HIV-1, which reactivate infectious virus production from viral latency in response to autocrine/paracrine S100A8-mediated glycolysis.

## Introduction

Current HIV-1 combination antiretroviral therapy (cART) is neither curative nor eradicates infection largely due to the establishment of cell reservoirs for the virus, which persistently shelter HIV-1 through poorly understood mechanisms. Apart from classically described CD4^+^ T-cell reservoirs, HIV-1 also establishes and persists in tissue macrophages^1,2^ by integrating viral genome^3^ into macrophage DNA. Viral genome integration promotes viral replication for weeks post-infection with limited viral lytic egress^3–5^, and finally resulting in latent macrophage infection^6^.

The latency of macrophage infection, i.e., the reversibly nonproductive state of infection^7^ in the weeks following infection has been demonstrated in vitro by restoring viral production of infected macrophage after activation of Toll-like receptor 4 (TLR4), the lipopolysaccharide (LPS) receptor^8^ by LPS. Accordingly, LPS is known to reactivate, once silent, integrated proviral DNA^9–11^. In chronically or latently infected macrophages, TLR4 engagement induces a signal transduction pathway that culminates in the activation of the nuclear factor-kappa B (NF-κB)^11^, which in turn efficiently triggers HIV-1 long-terminal repeat (LTR) promoters and HIV-1 replication^10^.

There is growing evidence that macrophages are directly involved in the persistence of HIV-1 in tissues^12–14^, keeping HIV-1 transcriptionally active despite cART^7^. Similar to tissue-like macrophages infected in vitro^6^, infected macrophages detected in vivo exhibit pathogen-containing vacuoles also known as virus-containing compartments (VCCs). This structure is absent from infected CD4^+^ T cells that are unable to store viral particles. In macrophages, VCCs might serve as storage compartments for infectious virus produced despite cART and prone to be transferred to other target cells upon external stimulation during cART^15^ and/or once antiretroviral therapy is interrupted^16^. We have previously shown that tissue-resident macrophages might constitute an important replication-competent HIV-1 reservoir^12^. This macrophage reservoir most likely forms following early tissue-macrophage infection during HIV-1 sexual transmission^6,17^. In vivo, the degree of plasticity of macrophages is much higher than in vitro. Hence, the two main polarization states in which macrophage can be differentiated in vitro are the pro-inflammatory M1 and the pro-reparative M2 macrophage subtypes. In contrast in vivo, CD68^+^ macrophages from HIV-1-infected cART-suppressed individuals harbor mainly a mixed M1/M2 phenotype expressing the IL-1 and IL-4 receptors, and CD206 but not the M2 marker CD163. As HIV chronic infection is associated with low-level chronic inflammation in the circulation but also in mucosal tissue, such a mixed M1/M2 phenotype could correspond to the recently described inflammatory M4-macrophage subtype that contributes to atherosclerosis. This M4-macrophage subtype is characterized by the expression of CD68, CD206, the metalloproteinase 7 (MMP7), and the calcium-binding protein A8 (S100A8) and M4 polarization is dependent on CXCL4/platelet factor 4 (PF4 )^18–21^. However, the precise characteristics of macrophage reservoirs at the single-cell level, their capacity to remain transcriptionally active despite cART, and the mechanism supporting the persistence of HIV-1 remain elusive.

HIV persistence mechanisms profoundly differ between T cells and macrophages. In the latter, it is tightly related to innate immunity inflammatory stimuli^22^. Furthermore, macrophages are key players in tissue homeostasis and reactive to metabolic adaptations potentially implicated in HIV-1 persistence^23–25^. We thus investigated whether mucosal macrophage HIV-1 reservoirs express inflammatory factors controlling reservoir maintenance, and whether in these reservoirs, macrophage metabolism could rule HIV-1 dynamics.

Here, we reveal that a specific macrophage subset, the inflammatory M4 one, is prone to glycolysis and constitutes the principal viral mucosal reservoir. When facing an inflammatory stimulus such as that induced by the inflammatory alarmin S100A8 expressed in particular by tissue M4-macrophages and which can target TLR4, the M4-macrophage metabolic profile shifts toward glycolysis. Furthermore, S100A8 maintains M4-macrophage reservoir persistence in an autocrine/paracrine loop by reactivating the production of replication-competent viral particles in male genital tract macrophages obtained from cART-suppressed individuals in a process controlled by a glycolytic metabolic adaptation.

The characterization of inflammatory M4-macrophages as the major HIV-1 reservoir in the genital mucosa from cART-suppressed individuals, together with the demonstration that macrophage glycolytic immunometabolism participates in HIV-1 latency reversal by controlling the production of infectious HIV-1 from these reservoirs, contribute to define novel pharmacological targets for a functional HIV-1 cure.

## Results

### Inflammatory macrophages with an M4 (S100A8^+^MMP7^+^) phenotype are enriched in the genital mucosa of cART-suppressed HIV-1-infected individuals

**Fig. 1:**
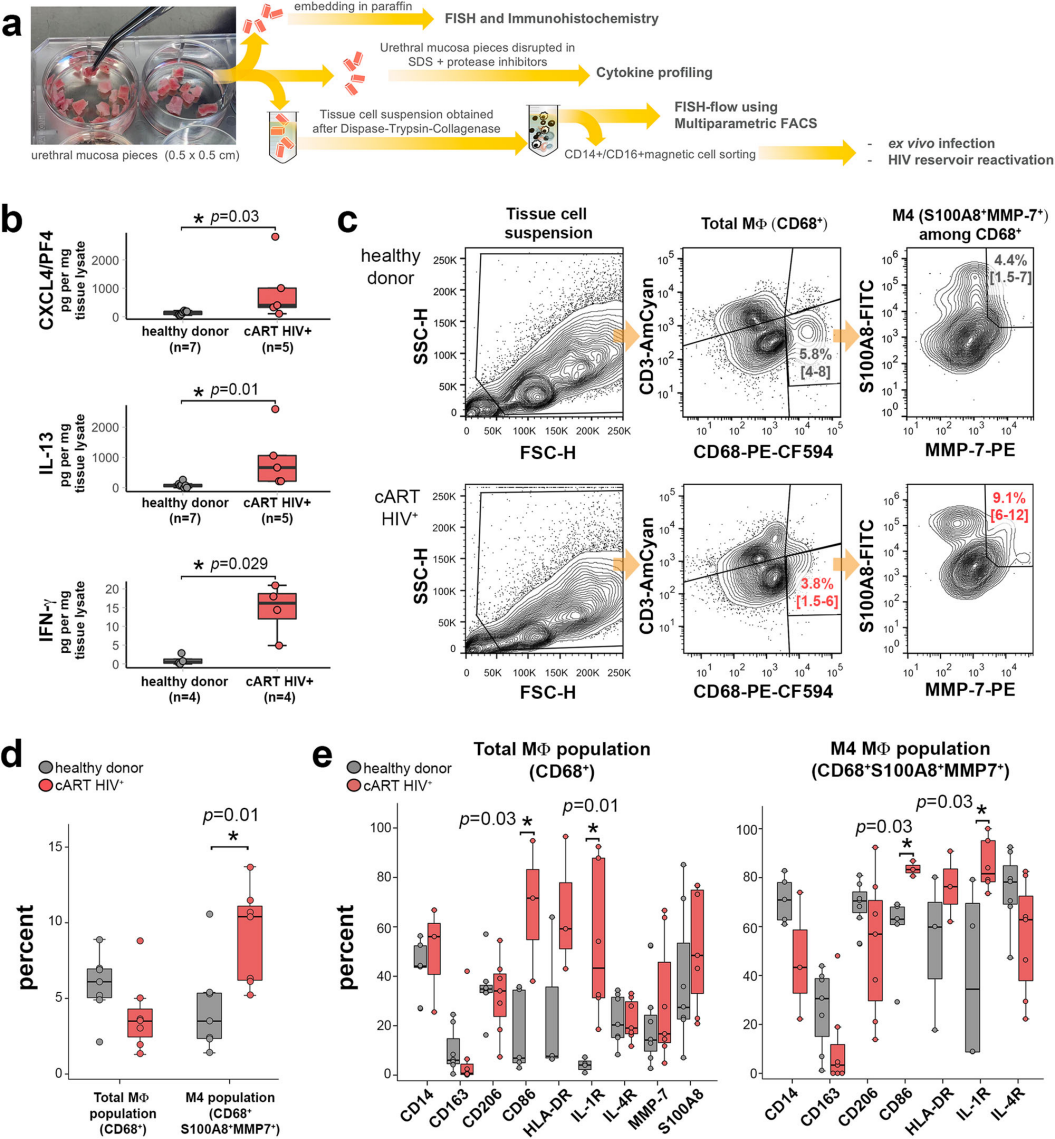
Inflammatory M4-macrophages are enriched in the urethral mucosa of cART-suppressed HIV-1-infected individuals. **a** Scheme of the different methods applied to evaluate morphologically and functionally the mucosal macrophage HIV reservoir, from processing 0.5 × 0.5 cm mucosal tissue pieces obtained from individuals that underwent gender reassignment. **b** CXCL4/PF4, IL-13, and IFN-γ cytokine profile of urethral tissue extracts of healthy donors (gray) and cART-suppressed HIV-infected (cART HIV^+^) individuals (red). The number of individual samples (*n*) included in the analysis is shown per group and cytokine tested. Mann–Whitney test performed for pairwise comparisons between the two groups of individuals and per cytokine. Source data are provided in Source Data file. **c** Flow cytometry gating strategy for characterization of M4-macrophage subtype in healthy donors (upper example) and cART-suppressed HIV-infected individuals (lower example). From CD68^+^ CD3^neg^ events (total MΦ), M4-macrophages are defined as S100A8^+^MMP7^+^ events. Frequencies of total MΦ and of M4-macrophages among total MΦ are shown as mean with 95% confidence interval in brackets. **d** Percentage of total MΦ among the tissue cell suspension (Total MΦ population) and percentage of M4-macrophages among the total MΦ population (M4 MΦ in total MΦ) in healthy donors (gray, *n* = 7) and cART-suppressed HIV-infected individuals (red, *n* = 7). Mann–Whitney test between healthy donor and cART HIV^+^ group. Source data are provided in Source Data file. **e** Frequency in percentage of macrophage polarization markers displayed by total MΦ among the tissue cell suspension (Total MΦ population, left graph) and M4-macrophages (M4-macrophages population, right graph) in healthy donors (gray, *n* = 7) and cART-suppressed HIV-infected individuals (red, *n* = 7). Mann–Whitney test between healthy donor and cART HIV^+^ group. Source data are provided in Source Data file.

We previously identified the TLR4 ligand LPS as an agent capable of reactivating replication-competent HIV-1 from genital tissue suspension. This indicated that tissue macrophages might form major replication-competent HIV-1 reservoirs in these mucosa^6,12^, although the precise identity of these reservoirs remained unclear. We have assessed mucosal macrophages from urethral tissue from healthy and HIV-infected cART-treated individuals (Table 1) by a series of experiments using various techniques as summarized in Fig. 1a. First, to determine the distinctive phenotype of HIV-1 reservoirs formed in tissue-resident macrophages at the single-cell level, we profiled the cytokines implicated in the polarization of different macrophage subtypes^26,27^ in urethral tissue lysate samples from healthy donors and cART-suppressed HIV-1-infected individuals comparatively, using the Luminex technology. C-X-C motif chemokine 4/platelet factor 4 (CXCL4/PF4), interleukin-13 (IL-13), interferon-γ (IFN-γ), and granulocyte-macrophage colony-stimulating factor (GM-CSF) were upregulated in mucosal tissue from cART-suppressed HIV-infected individuals as compared with non-infected donors (Fig. 1b and Supplementary Fig. 1a). CXCL4/PF4 directly correlated with IL-13 independently of the HIV status. However, only in the cART HIV^+^ group, CXCL4/PF4 correlated directly with IFN-γ and inversely with IL-4 (Supplementary Fig. 1b). The upregulation of CXCL4/PF4 in cART-suppressed HIV-infected individuals compared with healthy donors was confirmed by immunohistochemistry (Supplementary Fig. 2).

**Table 1:** Clinical information of HIV-infected cART-suppressed individuals enrolled in this study

Individual	Age at tissue sampling	Years of HIV diagnostic	Years of cART initiation	Viral load at sampling	cART regimen^a^
1	30	3	NA	Undetectable	NA
2	50	NA	NA	Undetectable	NA
3	43	22	NA	Undetectable	RTV DRV RAL ETR
4	38	23	NA	Undetectable	FTC TDF RTV
5	38	NA	NA	Undetectable	NA
6	53	NA	11	Undetectable	FTC TDF EVG
7	59	NA	NA	Undetectable	FTC TDF EVG
8	51	4	NA	Undetectable	3TC DTG ABC
9	34	3	3	Undetectable	FTC TDF EVG
10	40	14	11	Undetectable	FTC TDF EVG
11	41	NA	NA	Undetectable	NA
12	36	NA	NA	Undetectable	NA
13	59	13	13	Undetectable	DTG RPV
14	26	9	9	Undetectable	FTC TDF EFV
15	37	8	NA	Undetectable	FTC TDF EFV
16	35	16	9	Undetectable	FTC TDF RTV DRV
17	32	2	2	Undetectable	NA
18	45	18	18	Undetectable	3TC ABC LPV RTV
19	45	4	4	Undetectable	FTC TDF RTV DRV
20	47	28	10	Undetectable	FTC TDF RPV
21	36	10	2	Undetectable	3TC DTG ABC
22	65	4	4	Undetectable	NA
23	49	24	22	Undetectable	3TC DTG
24	44	15	14	Undetectable	FTC TDF RPV
25	51	NA	NA	Undetectable	NA

The table shows the age, years after HIV-positive diagnosis, and years under combined antiretroviral therapy (cART) of the individuals at the date of tissue sampling. The viral load of these individuals was tested undetectable at the date of sampling (HIV RNA copies/mL of blood below the limit of detection of 40 copies/mL). All individuals were receiving different suppressive cART regimens at sampling, although not all regimens were fully disclosed (not available, NA).

^a^cART includes nucleoside reverse transcriptase inhibitors (3TC, lamivudine; ABC, abacavir; FTC, emtricitabine; TDF, tenofovir), non-nucleoside reverse transcriptase inhibitors (EFV, efavirenz; ETR, etravirine; RPV, rilpivirine), protease inhibitors (ATV, atazanavir; DRV, darunavir; FPV, fosamprenavir; RTV, ritonavir; LPV, lopinavir), and integrase inhibitors (DTG, dolutegravir; EVG, elvitegravir; RAL, raltegravir); NA, not available.

IL-13 is implicated in the alternative activation of macrophages resulting in a pro-reparative CD206^+^CD163^+^ macrophage subset that antagonizes IFN-γ inflammatory actions and is implicated in humoral immunity, allergies, and anti-parasitic responses^28^. The participation of bona fide pro-reparative CD163^+^ macrophages in HIV-1 tissue reservoirs could be discarded based on our recent results showing that CD163^neg^ but CD206^+^IL4-R^+^IL1-R^+^ macrophages with an intermediate pro-inflammatory/pro-reparative profile are enriched in urethral tissue of cART-treated patients^12^, and are the likely site of HIV persistence in the male genital mucosa. However, IL-13 can also drive macrophage expression of DC-SIGN, an alternative receptor for HIV-1, thus rendering macrophages highly susceptible to HIV infection. The upregulation of IL-13 in mucosal tissues from cART-suppressed HIV-1-infected patients we observed might result from the immunomodulatory activity of CXCL4/PF4 on Th2 lymphocytes^29^.

Furthermore, CXCL4/PF4 is mainly expressed by cells of the megakaryocyte lineage and consequently in platelets but also by some epithelial cells^26,30^. One function of CXCL4/PF4 is to induce in vitro the polarization of macrophages toward the recently described M4 subset^31^, an inflammatory macrophage subset related to foam cell formation, atherosclerosis, and intracellular infection^26,30^. M4-macrophages express inflammatory surface markers such as HLA-DR and CD86 but lack CD163^26^. Such macrophage profile matches that of CD163^neg^ but CD206^+^IL4-R^+^IL1-R^+^ activated macrophage subtype we have found implicated in mucosal persistence of HIV-1^12^. M4-macrophages are defined, both in vitro and in vivo, by the co-expression of the S100A8 and S100A9 also known as Myeloid-related protein 8 and 14 (MRP8 and MRP14) or Calgranulin A^30,32^, along with MMP7^33^. The markers CD68^+^S100A8^+^MMP7^+^ thus identify M4-macrophages.

To search for M4-macrophage in urethral tissues in HIV/AIDS, we analyzed the frequency of total mucosal (CD68^+^) macrophages in urethral cell suspensions and therein, the frequency of M4-macrophages (S100A8^+^MMP7^+^) by flow cytometry (Fig. 1c, d and Supplementary Fig. 3). Although the frequency of macrophages did not differ between healthy donors and cART-suppressed HIV-infected individuals, M4-macrophages were enriched in urethral tissues from the HIV-1-infected group compared with healthy donors (Fig. 1c, d). The expression of common markers of macrophage polarization in total urethral macrophages (CD68^+^) and in M4-macrophages was also investigated. We observed an increased frequency of macrophages expressing the inflammatory markers CD86 and IL-1R among both total macrophages (Fig. 1e left for frequencies and Supplementary Fig. 3d, left for MFI) and M4-macrophages (Fig. 1e right for frequencies and Supplementary Fig. 3d, right for MFI) from cART-suppressed HIV-1-infected individuals compared with healthy donors.

### Inflammatory IL-1R^+^ M4-macrophages are the main HIV-1 mucosal macrophage reservoirs in vivo

**Fig. 2:**
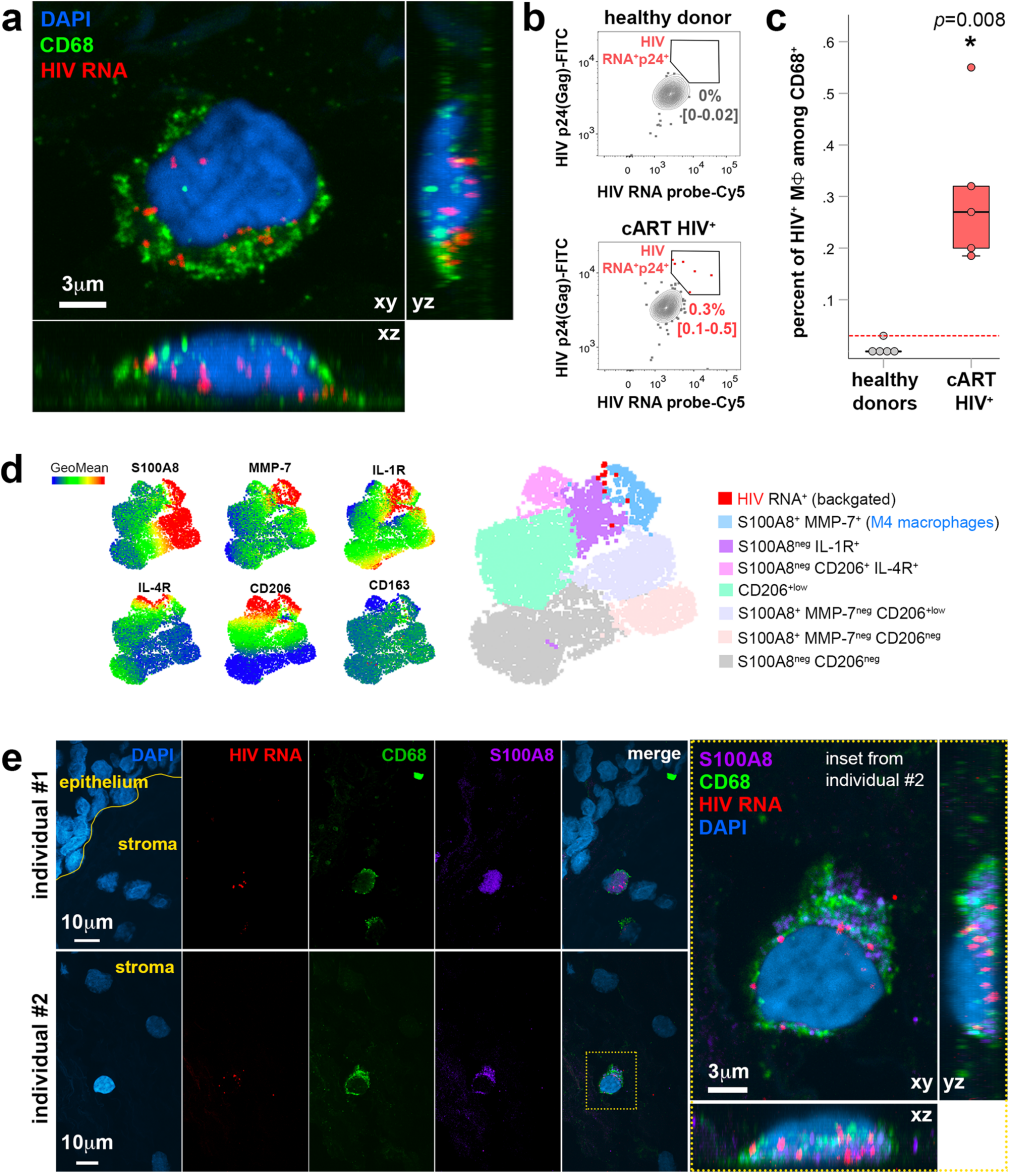
HIV-1 mucosal macrophage reservoirs form in inflammatory IL-1R+ M4-macrophages in vivo as detected at single-cell level. **a** Identification of HIV reservoir in macrophages identified by HIV RNA detection using FISH coupled to CD68 immunostaining on paraffin-embedded urethral tissue sections from a cART-treated HIV-infected individual. HIV RNA in red, macrophage marker CD68 in green, nuclei stained with DAPI (blue). Image shows the different fluorescent channels of a macrophage HIV reservoir and a high-magnification inset on the right with these channels merged. Bar = 3 μm (inset). Image is representative of two cART-treated HIV-infected individual samples. **b** Representative flow cytometry gating of HIV RNA^+^p24-Gag^+^ events acquired in total urethral MΦ population obtained from a healthy donor (upper) and a cART-suppressed HIV-infected individual (lower). Frequencies of HIV RNA^+^/p24-Gag^+^ MΦ among total MΦ are shown as mean with 95% confidence interval in brackets (*n* = 5 healthy donors, *n* = 5 cART HIV-infected individuals). **c** Percentage of HIV RNA^+^p24-Gag^+^ events acquired in total urethral MΦ population obtained from these two groups of individuals (*n* = 5 healthy donors, *n* = 5 cART HIV-infected individuals). Dotted red line indicates limit of detection based on healthy donor controls. Mann–Whitney test. Source data are provided in Source Data file. **d** UMAP built from concatenating CD68^+^ cell populations of different cART HIV^+^ individuals (*n* = 4) after clustering analysis of macrophage populations. HIV^+^ macrophages were backgated into the UMAP to identify which macrophage profile harbors HIV in cART-treated individuals. **e** S100A8 is expressed by HIV reservoir macrophages as shown by colocalization of HIV RNA detected using FISH coupled to CD68 and S100A8 immunostaining on paraffin-embedded urethral tissue sections from two cART-treated HIV-infected individuals (#1 and #2). HIV RNA in red, macrophage marker CD68 in green, S100A8 in magenta, nuclei stained with DAPI (blue). Image shows the different fluorescent channels (separated and merged) of the detected macrophage HIV reservoirs found in the mucosal stroma. A high-magnification inset for the one detected in individual #2 is shown on the right, with fluorescent channels merged. Bar = 10 or 3 μm (inset).

The macrophage HIV reservoirs found in the urethral mucosal stroma from cART-suppressed individuals are a transcriptionally active reservoir in which HIV RNA was detected by fluorescent in situ hybridization (FISH) (Fig. 2a). We quantified HIV-1-infected macrophages in urethral tissues from cART-suppressed individuals at the single-cell level combining FISH and flow cytometry referred to as FISH-flow^34,35^ to detect cell-associated HIV together with markers of different macrophage profiles, including the M4-defining S100A8 and MMP7. We first validated the FISH-flow HIV-1 RNA probes using OM-10.1 cell lines (Supplementary Fig. 4a, b) which constitutively harbor integrated HIV provirus and can be stimulated to produce more HIV-1 upon TNF-α stimulation^36^.

In HIV-1-infected individuals despite cART-suppression, we detected a subpopulation of macrophages containing HIV-1 RNA and the viral capsid protein p24 (Gag) that represents 0.3% [CI: 0.12–0.48] of total mucosal macrophages present in tissue cell suspensions (Fig. 2b, c and Supplementary Fig. 4c).

To characterize these HIV^+^ macrophages, we generated uniform mainifold approximation and projections (UMAP) from the multiparametric flow cytometry analysis including S100A8, MMP7, IL-1R, IL-4R, CD206, and CD163 markers, and compiled data from 4 cART HIV^+^ individuals. The rare HIV^+^ macrophages detected were backgated into this UMAP (Fig. 2d). The results showed that HIV^+^ macrophages were distributed in the M4 population (light blue population in UMAP, S100A8^+^MMP7^+^CD206^+^IL-1R^+^) and in a second population (purple population in UMAP) characterized by S100A8^neg^MMP7^+^CD206^+^IL-1R^+^ macrophages. Around half of the infected macrophages (52.7% [CI: 16.6–100]) belonged to the S100A8^+^MMP7^+^ M4-macrophage profile (Supplementary Fig. 4d). Furthermore, these HIV-1-infected macrophages expressed also CD206, IL-4R, and IL-1R but not CD163 (Fig. 2d and Supplementary Fig. 3d), in agreement with the previously uncharacterized CD206^+^IL4-R^+^IL1-R^+^CD163^neg^ intermediary subset of macrophages we found enriched in the male urethra of cART-suppressed individuals^12^. Finally, S100A8 was expressed by the macrophage HIV reservoirs as demonstrated by the immunodetection of S100A8 within HIV-1 reservoirs identified as CD68^+^/HIV-1 RNA^+^ cells using combined immunohistochemistry and RNAscope FISH (Fig. 2e).

Altogether, we have identified a transcriptionally active (HIV-1 RNA^+^) reservoir formed mainly in M4-macrophages that express the pro-inflammatory activation marker IL-1R and, importantly, the alarmin S100A8.

### In vitro derived and latently infected M4-macrophages are surrogates of HIV-1 mucosal macrophage reservoirs

As mucosal tissues from HIV-1-infected individuals are rare, hampering the characterization of HIV reservoir dynamics, we established alternative models of HIV-1 reservoir in macrophages, either from monocytes or from tissue macrophages infected in vitro until latency self-establishment.

**Fig. 3:**
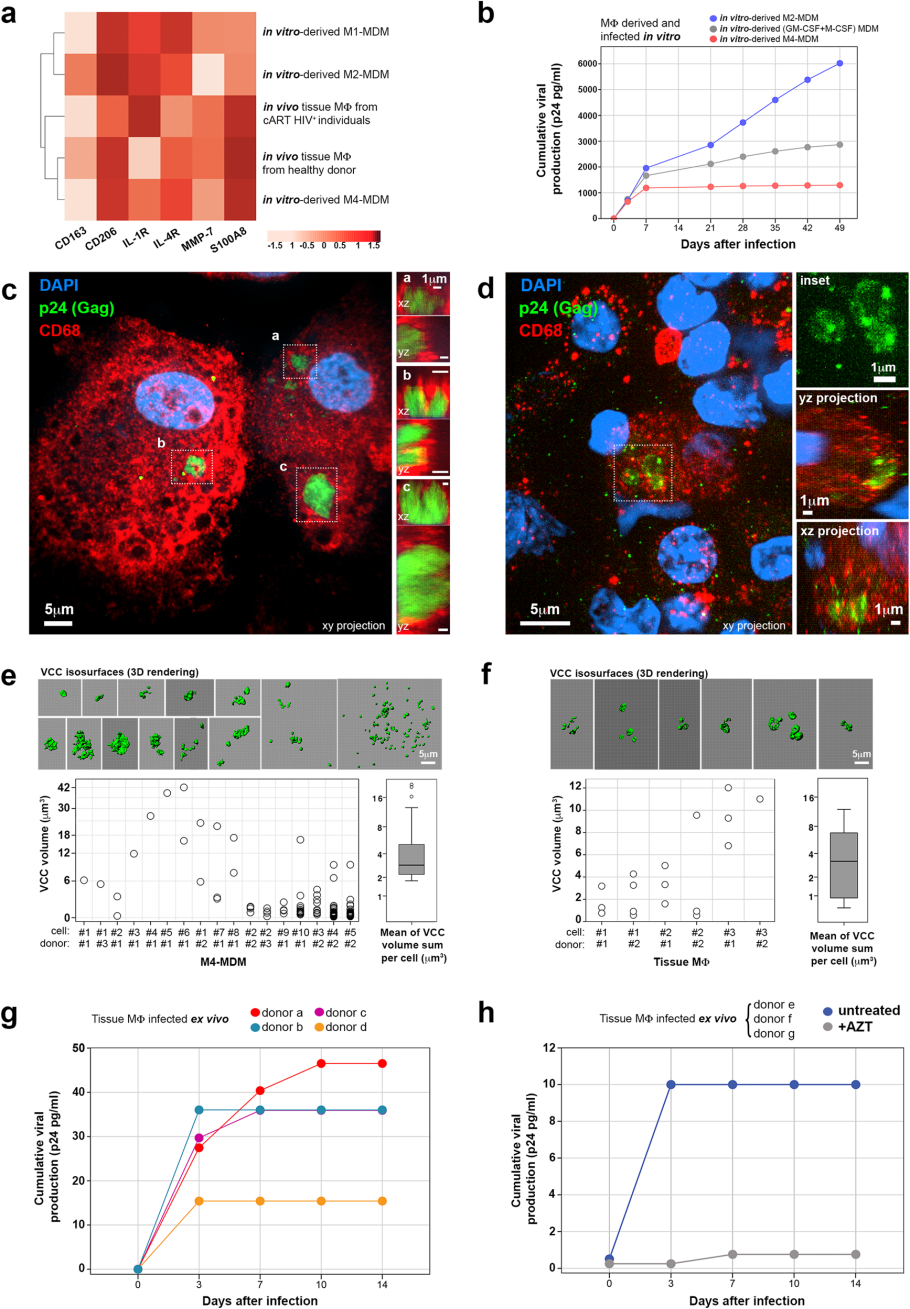
M4-MDM and tissue macrophages from healthy donors infected in vitro/ex vivo are surrogates of HIV-1 mucosal macrophage reservoirs. **a** Hierarchical clustering heatmap of macrophage polarization markers and different macrophage subtypes (obtained in vivo or in vitro derived by different stimuli). The intensity of the red color boxes refers to correlation *z*-scores. Data were obtained from four independent experiments. Source data are provided in Source Data file. **b** Cumulative viral production assessed in in vitro-derived MΦ culture supernatants on different days after infection. M2-MDM in blue, non-polarized GM-CSF/M-CSF-derived MDM in gray, and M4-MDM in red. Data refer to one experiment representative of three independent experiments. Source data are provided in Source Data file. **c**, **d** Confocal microscope images of M4-MDM latently infected in vitro (34 days post-infection) (*n* = 3) (**c**) and infected tissue macrophage obtained from cART-suppressed HIV-infected individuals (*n* = 2) (**d**), immunolabeled for p24-Gag (green) and CD68 (red). Nuclei were stained with DAPI. Merged images are shown as xy projections (main images) and xz/yz projections (framed VCC *a*, VCC *b* and VCC *c* in C right insets, and in D, lower right insets). In D, VCCs framed and magnified in the upper-right inset showing p24-Gag^+^ fluorescence signal only. Bar = 1, 5, or 10 μm. **e**, **f** Volumetric morphometry of VCCs in infected macrophages obtained from M4-MDM latently infected in vitro (*n* = 3) (**e**) or cART-suppressed HIV-infected tissues (*n* = 2 individuals) (**f**) after image segmentation in VCC isosurfaces. Dot plots show the volumes (μm^3^) of each VCC isosurface detected in different macrophages (identified by number #) obtained from different donors (identified by donor number #). Boxplots represent the sum of VCC volumes per macrophage. Upper panels show VCC isosurfaces (in green, 3D rendering) segmented from the confocal images of infected macrophages. Source data are provided in Source Data file. **g** Cumulative viral production assessed in the supernatants of tissue macrophages obtained from healthy donors (discriminated by different colors, *n* = 4) and infected ex vivo on different days after infection. Source data are provided in Source Data file. **h** Cumulative viral production measured in the supernatants of ex vivo-infected urethral macrophages (pool of three healthy donors) in the absence or presence of 10 μM AZT. Source data are provided in Source Data file.

M4-macrophages can be differentiated from blood monocytes in vitro by culture in the presence of CXCL4/PF4^30,32^, resulting in M4-monocyte-derived macrophages (MDM). We first investigated whether M4-MDM would be surrogates of the tissue macrophages found in the urethral mucosa by flow cytometry. Relative to the expression of tested macrophage surface markers, especially S100A8, we found that M4-MDM were more closely related to the population of urethral macrophages isolated either from HIV-1-infected or healthy individuals than other in vitro derived macrophage subtypes, namely “classically” activated pro-inflammatory M1-MDM (induced by GM-CSF, IFN-γ and LPS) and alternatively activated pro-reparative M2-MDM (induced by M-CSF, IL-4, and IL-13) (Fig. 3a).

When infected with HIV-1, the different MDM subsets were productively infected. At later time points, whereas non-polarized-MDM induced by GM-CSF/M-CSF or M2-MDM, although at a lower level in agreement with others^3,24^, continue both to produce viral particles until day 49 after infection as detected by p24 enzyme-linked immunosorbent assays (ELISA), M4-MDM reduced viral production and enters latency characterized by undetectable viral secretion from day 8.5 [CI: 7–15] (Fig. 3b).

At the morphological level, M4-MDM harbored viruses in VCCs (Fig. 3c) during the latent phase of viral production, i.e., when macrophages stop producing new viral particles into the culture medium. Similarly, in mucosal tissues CD68^+^ macrophages sheltered HIV-1 in VCCs as detected by p24 labeling (Fig. 3d). We next performed morphometric analyses of VCCs formed in vitro by latently infected M4-MDM (Fig. 3e) and formed in vivo by mucosal macrophages in tissues (Fig. 3f). These analyses revealed that although VCCs in M4-MDM were more heterogeneous in size, number and scattering, the sum of the VCCs volume per infected macrophage did not differ from that in infected mucosal tissue macrophage reservoirs formed in vivo. A panel of additional macrophage VCCs detected in vivo in tissues is shown in Supplementary Fig. 5a.

Finally, we also infected genuine mucosal tissue macrophages purified from healthy donor tissue with HIV-1 in vitro. Tissue macrophages were productively infected by HIV-1 in a similar manner to M4-MDM, but established latency even earlier, reaching latency after a median interval of 5 [CI: 3–10] days post-infection (Fig. 3g) compared with 8.5 [CI: 7–15] days post-infection for M4-MDM. Viral production by ex vivo-infected urethral macrophages is abrogated by the antiretroviral drug Zidovudine (AZT), confirming productive infection (Fig. 3h).

We thus established two reliable surrogates of the tissue macrophage reservoirs found in urethral mucosa, formed by M4-MDM, and tissue macrophages acutely infected in vitro by HIV-1 and maintained at least for a week in culture.

### S100A8 can reactivate the production of replication-competent virus from macrophage reservoirs

S100A8 is always expressed together with S100A9 as a heterodimer^37^. However, to show the full activity of the S100A8/S100A9 complex, it is necessary to use S100A8 homodimers in cell culture studies because the activity of the heterodimer is very low in vitro due to the formation of heterotetramers that blocks the S100A8/S100A9 complex activity^37^.

S100A8/S100A9 activates specifically TLR4^38^, which in turn can reactivate HIV-1 production^39^. We thus hypothesized that formation and persistence of HIV-1 reservoirs in M4-macrophages is regulated by a local S100A8/S100A9-mediated low-level HIV-1 production in genital tissues. When secreted in tissues, mainly from neutrophils or macrophages, S100A8/S100A9 forms rapidly heterotetramers^37^, thereby buffering its activity. It might remain nevertheless a short window of time during which the S100A8/S100A9 heterodimer could be active locally in an autocrine/paracrine fashion.

**Fig. 4:**
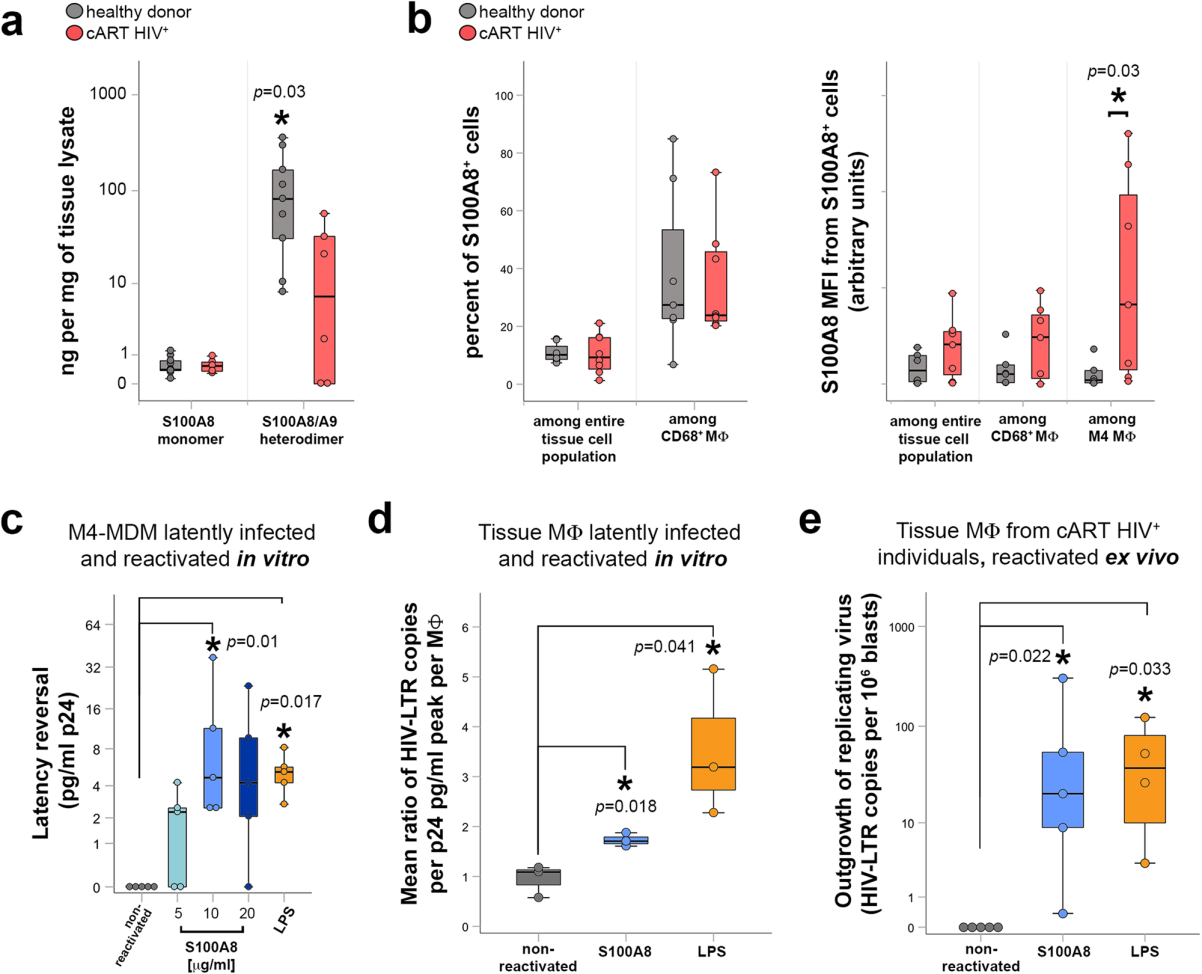
S100A8 reactivate the production of replication-competent virus from tissue macrophage reservoirs. **a** Quantification of S100A8 monomers and S100A8/S100A9 heterodimers in urethral tissue extracts of healthy donors (gray, *n* = 9) and cART-suppressed HIV-infected individuals (cART HIV^+^) (red, *n* = 6). Mann–Whitney test between healthy donor and cART HIV^+^ group. Source data are provided in Source Data file. **b** S100A8 cellular expression quantified by flow cytometry of urethral cell suspensions expressed as percentage of S100A8^+^ cells among the entire tissue cell population and among CD68^+^ MΦ, or as S100A8 Mean Fluorescence Intensity (MFI) in S100A8^+^ cells detected among the entire tissue cell population, among CD68^+^MΦ, or among M4 MΦ. Mann–Whitney test between healthy donor (*n* = 5) and cART HIV^+^ group (*n* = 7). Source data are provided in Source Data file. **c** Viral production quantified in M4-MDM culture supernatants after latent infection followed by 2 days of latency reversal induced by indicated S100A8 concentrations or 1 μg/mL LPS. Non-reactivated controls refer to untreated M4-MDM latently infected macrophages. Mann–Whitney test between non-reactivated (untreated) and treated groups pairwise. Data were obtained from five independent experiments. Source data are provided in Source Data file. **d** HIV-1 latency reversal of tissue macrophages obtained from healthy donors latently infected ex vivo (*n* = 3), expressed as ratio between macrophage-associated HIV-LTR RNA copies per MΦ and the peak cumulative viral production per MΦ before latency, after 2 days of S100A8 or LPS treatment. Non-reactivated controls refer to ratio from untreated latently infected tissue macrophages. Mann–Whitney test between non-reactivated (untreated) and treated groups pairwise. Source data are provided in Source Data file. **e** Outgrowth of replicating virus produced by tissue macrophage reservoirs obtained from cART-suppressed HIV-infected individuals after S100A8- or LPS-induced latency reversal, expressed as HIV LTR RNA copies per million CD4^+^ T-cell lymphoblasts from the outgrowth assays. Non-reactivated controls refer to untreated tissue macrophage reservoirs. Pairwise Mann–Whitney test between non-reactivated (untreated, *n* = 5) and treated groups (S100A8, *n* = 5; LPS, *n* = 4). Source data are provided in Source Data file.

We thus measured the levels of S100A8 monomer and S100A8/S100A9 heterodimers in urethral tissues by ELISA. S100A8/S100A9 heterodimer, the physiologically relevant form, was expressed at a much higher concentration than the S100A8 monomer, as expected. Furthermore, the S100A8/S100A9 heterodimer concentration was significantly reduced in cART-suppressed HIV-1-infected individual as compared with healthy donor tissues (Fig. 4a), in line with increased neutrophil cell death observed in HIV-infected individuals on cART^40^.

We further investigated whether the measured tissue S100A8 and its expression at the tissue macrophage level were associated. When analyzed by flow cytometry, the frequency of S100A8^+^ cells remained similar in both healthy donors and HIV-1-infected individuals. However, the expression of S100A8 per cell, corresponding to the MFI, was specifically upregulated in M4-macrophages isolated from cART-suppressed HIV-1-infected individuals compared with tissue M4-macrophages from non-infected individuals but also with cells from the entire tissue cell population and total tissue macrophage population of infected and non-infected individuals (Fig. 4b). Therefore, although the total mucosal tissue S100A8/S100A9 heterodimer level was decreased in cART-suppressed infected individuals, the level of S100A8 was higher in M4-macrophages from cART-suppressed infected individuals, the main macrophage subset that host HIV-1, compared with healthy donor ones. In tissue from cART-treated individuals that are subjected to chronic low-level inflammation^41^, S100A8 could be locally secreted upon M4-macrophage stimulation and act in an autocrine/paracrine manner to reactivate the macrophage reservoirs upon binding to TLR4.

To investigate the capacity of S100A8 to reverse HIV-1 latency in macrophage reservoirs, we first evaluated whether S100A8 treatment would reverse latency in HIV-1-infected M4-MDM. For these cell culture-based studies, we used the homodimer S100A8 instead of the physiological relevant heterocomplex S100A8/S100A9 due to the rapid loss of activity by heterotetramerization^37^. S100A8 resumed viral production from an HIV-1 latent state in M4-MDM (Fig. 4c) in a concentration-dependent manner from 5 to 20 μg/mL, as measured by p24 ELISA. Latency reversal by S100A8 was as efficient as that induced by LPS at a concentration of 1 μg/mL, a treatment we and others have already shown to reactivate HIV-1 production from macrophages latently infected by HIV in vitro or in vivo^6,12^. Moreover, in latently infected M4-MDM, HIV DNA was found integrated into the host cell genome, as measured by Alu-gag nested PCR (Supplementary Fig. 5b, left). The addition of S100A8 to latently infected M4-macrophages resumed viral transcription. S100A8-mediated viral reactivation is specific, as the antiretroviral drug AZT blocked it to the level prior to reactivation (Supplementary Fig. 5b, right).

Furthermore, S100A8 at 10μg/mL was also efficient at reactivating viral production from mucosal tissue macrophages isolated from healthy donors and latently infected by HIV-1 in vitro used as another HIV-1 macrophage reservoir surrogate (Fig. 4d).

Finally, S100A8 reversed latency in tissue macrophages isolated from cART-suppressed HIV-1-infected individuals. The reactivated virus was replication-competent as quantified in a viral outgrowth assay using activated CD4^+^ T cells (Fig. 4e). Additionally in tissues from cART-treated individuals, HIV-1 RNA in situ hybridization coupled to p24-Gag immunodetection in CD68^+^ macrophages further demonstrated that clusters of p24 (Gag) proteins co-localized with HIV-1 RNA, a pattern suggestive of VCCs before ex vivo S100A8 treatment (Supplementary Fig. 5c, upper). Upon S100A8-mediated HIV-1 reactivation, both HIV-1 RNA and protein relocalized at the macrophage plasma membrane that is suggestive of extemporaneous production of new HIV-1 particles and/or mobilization of VCCs to the macrophage plasma membrane in the process of releasing their virus content (Supplementary Fig. 5c, lower).

### S100A8-mediated reactivation of HIV-1 release from macrophage reservoirs depends on glycolysis

After engaging TLR4, microbial components such as LPS trigger a pro-inflammatory response in macrophages that was recently described to engage metabolic reprogramming, which contributes to macrophage plasticity and physiology^42^. As we have shown that mucosal macrophage HIV-1 reservoirs express pro-inflammatory markers (Figs. 1 and 2), we investigated whether S100A8-mediated reactivation of the mucosal macrophage reservoirs would trigger a similar metabolic reprogramming related to inflammatory responses.

**Fig. 5:**
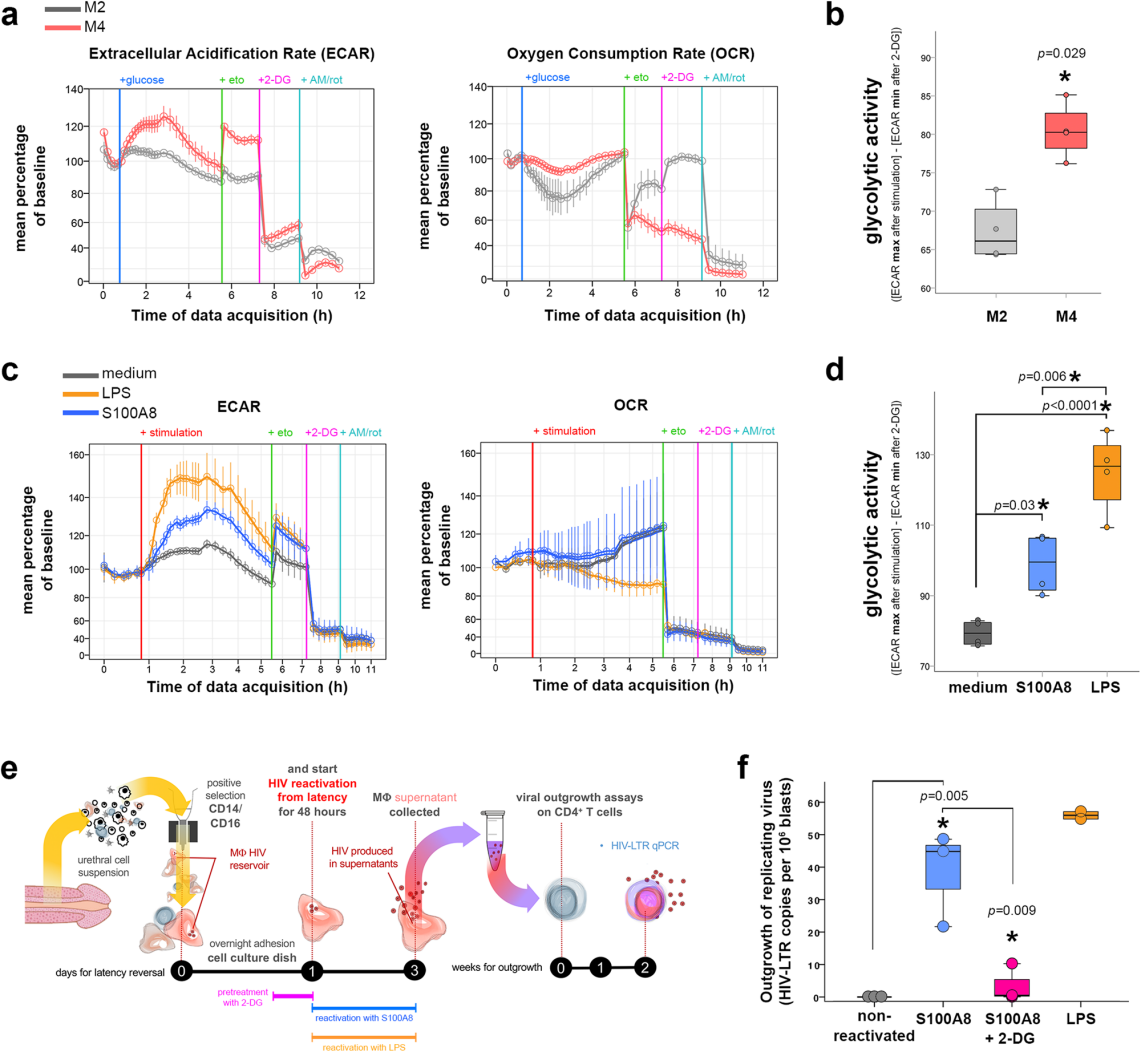
S100A8-mediated reactivation of HIV macrophage reservoirs depends on a glycolytic metabolic shift. **a** Extracellular acidification (ECAR) and oxygen consumption rate (OCR) of M2-MDM (gray) or M4-MDM (red) cultures measured after sequential addition of Seahorse medium with glucose, etomoxir (eto), 2-deoxyglucose (2-DG) and antimycin/rotenone (AM/rot). Results are shown as mean percentages (±SEM) of rate increase/decrease from baseline rate (time point before glucose injection) for each technical replicate. Data obtained from *n* = 4 independent experiments. Source data are provided in Source Data file. **b** Glycolytic activity of M2-MDM (gray) or M4-MDM (red) groups, which refers to the maximum ECAR reached after glucose injection deduced from the minimum ECAR reached after 2-DG injection. Mann–Whitney test. Data obtained from *n* = 4 independent experiments. Source data are provided in Source Data file. **c** ECAR and OCR measured in M4-MDM cultures non-stimulated (medium with glucose) or stimulated with S100A8 or LPS in the presence of glucose, followed by sequential injection of etomoxir (eto), 2-deoxyglucose (2-DG) and antimycin/rotenone (AM/rot). Results are displayed as mean percentages (±SEM) of rate increase/decrease from baseline rate (time point before glucose injection) for each technical replicate. Data obtained from *n* = 4 independent experiments. Source data are provided in Source Data file. **d** Glycolytic activity of non-stimulated and S100A8- or LPS-stimulated M4-MDM. ANOVA with Bonferroni post hoc test. Data obtained from *n* = 4 independent experiments. Source data are provided in Source Data file. **e**, **f** Outgrowth of replicating virus produced by tissue macrophage reservoirs obtained from cART-suppressed HIV-infected individuals after S100A8 latency reversal with or without 2-DG pretreatment. **e** Scheme of the experiment measuring the reactivation of macrophage HIV reservoirs obtained in vivo from urethral tissues ex vivo using the different stimuli, namely S100A8, S100A8 preceded by a 2-DG pretreatment, and LPS. The supernatants of reactivated macrophages were added to CD4^+^T cells in an outgrowth assay which readout is the detection of HIV RNA in the CD4^+^T cells by qPCR. **f** Outgrowth of virus produced by reactivated macrophage HIV reservoirs expressed as cell-associated HIV LTR RNA copies per million CD4^+^ T-cell lymphoblasts employed in outgrowth assays. Non-reactivated controls refer to untreated tissue macrophage reservoirs. ANOVA with Bonferroni correction. Data were obtained from three different cART HIV-infected individuals. Source data are provided in Source Data file.

The macrophage metabolic profile varies according to macrophage polarization from pro-reparative to pro-inflammatory macrophages. In “classically” IFN-γ/LPS activated M1-macrophages, aerobic glycolysis is induced, as well as glucose-induced repression of respiration, beneficial for aerobic glycolysis what is known as the Warburg effect in cancer cells^43^. “Alternative” IL-4/IL-13-activated M2-macrophages obtain energy preferentially from fatty acid oxidation and oxidative metabolism via oxidative phosphorylation (OXPHOS)^42^. Little is known about the metabolism of other alternative macrophage subsets implicated in inflammatory diseases such as the M4 subset^44^. We thus first investigated the metabolic profile of in vitro-derived inflammatory M4-macrophages compared with pro-reparative M2-macrophages. Therefore, M2- and M4-MDM basal metabolism was evaluated comparatively by measuring extracellular acidification rate (ECAR, to assess glycolysis) and oxygen consumption rate (OCR, to assess OXPHOS) upon injection of glucose into macrophage cultures. As a result, M4-MDM presented a mixed metabolic profile, carrying out glycolysis (inhibited by 2-deoxyglucose, or 2-DG) but also fatty acid oxidation (inhibited by etomoxir). Upon glucose addition to the culture medium, M4-MDM glycolytic activity was higher than that of M2-MDM (Fig. 5a, left) whereas M4-MDM OCR was only slightly repressed compared to that of M2-MDM (Fig. 5a, right). By adding metabolic inhibitors to macrophages during their metabolic evaluation, the glycolytic activity of these cells could be recorded. As a result, M4-macrophages had higher glycolytic activity than M2-macrophages (Fig. 5b). As compared with M2-MDM whose glycolytic activity remains close to that of baseline after glucose addition, M4 were more prone to glycolysis. Indeed, after the addition of glucose, M4-MDM glycolysis activity increased by 25% from baseline (Supplementary Fig. 6a). These data define M4-macrophages as a pro-inflammatory, glycolytic subset among the spectrum of different macrophage immunometabolic profiles.

Next, we evaluated the metabolic responses of M4-macrophages in glucose-supplemented medium after injection of medium alone (untreated), S100A8 (that we have shown above to act as HIV-1 latency reversal agent), or LPS. S100A8 triggered a significant glycolytic activity of M4-MDM, higher than that in the absence of stimulation, but less intense than the LPS-stimulated glycolysis (Fig. 5c, left and Fig. 5d). We observed an increase in glycolysis activity of ∼15%, ∼40%, and ∼60% from baseline after injection of medium alone (untreated), S100A8 and LPS, respectively (Supplementary Fig. 6b).

In contrast, S100A8 did not affect the OCR whereas LPS slightly decreased it (Fig. 5c, right). Altogether, LPS and S100A8 affected M4-macrophages metabolism by increasing mainly glycolysis with no major impact on cell mitochondrial respiration. These differences in the intensity of glycolytic activity in M4-macrophage upon stimulation by S100A8 and LPS may explain the difference in the intensity of latency reversal observed in M4-macrophage reservoirs treated with these two compounds.

We next evaluated whether latency reversal in HIV-1 macrophage reservoirs can be triggered upon glycolytic stimulation via TLR4. Therefore, in vivo established tissue macrophage reservoirs obtained from cART-suppressed HIV-1-infected individuals were treated with S100A8 to resume the production of replication-competent HIV-1 in the absence of or with pretreatment with the glycolytic inhibitor 2-DG as schematized in Fig. 5e. S100A8 triggered HIV latency reversal in tissue macrophage reservoirs resulting in infectious virus production in the culture medium, as quantified in the outgrowth assays using CD4^+^T cells as reporter cells (Fig. 5f). Inhibition of glycolysis before induction of latency reversal by S100A8 fully blocked the reactivation of replication-competent viral production by macrophages (Fig. 5f). Altogether, immunometabolic glycolytic pathways control HIV-1 reservoir reactivation in tissue macrophages.

## Discussion

The characterization of the degree of differentiation/polarization toward a specific macrophage subtype capable of hosting HIV-1 in human mucosal tissues permits to identify a central host cell therapeutic target against the virus in male genital mucosa. Macrophages are known to be extremely versatile cells, able to respond to a plethora of factors by differentiating into functionally distinct subsets. Each tissue-specific environment, presenting a characteristic set of cytokines, could drive macrophages toward HIV-1-resistant or -susceptible states, not necessarily associated with the in vitro established M1/M2 macrophage polarization paradigm^3,27^. Our study is the first, to our knowledge, to measure macrophage-related cytokines in the urethral mucosa comparing healthy donors and HIV-infected individuals under cART. Despite effective blood viral suppression by cART, HIV-infected individuals present persistent chronic immune activation, albeit at low levels in blood and tissues^41,45,46^. Resulting changes in microenvironment could polarize macrophages toward specialized profiles implicated in viral persistence.

Here, we demonstrated that in the urethral tissue from cART-suppressed HIV-infected patients, the HIV-1 replication-competent reservoir resides in a recently described subtype of inflammatory macrophages classified as M4. M4 polarization is mainly induced by CXCL4/PF4^18–21^ that we have found upregulated in the urethral mucosa of cART-suppressed HIV^+^ individuals as compared with healthy donors. Macrophages in most tissues are derived from embryonic progenitors, displaying a long life span and self-renewal capacities, and blood monocytes contribute only minimally to the pool of tissue macrophages under homeostatic conditions^47^. However, additionally, persistent tissue inflammation as commonly observed in HIV infection^48^ might also contribute to reservoir establishment and persistence. Such persistent tissue inflammation would favor the entry and integration of monocytes in the resident macrophage pool in tissues^47^, likely impacting viral reservoir dynamics. Stable tissue HIV reservoirs would persist in long-lived tissue macrophages, likely formed upon their early infection during sexual transmission^6,17^. In addition, inflammatory macrophages derived from blood monocytes^47^ might also either replenish the macrophage reservoir by becoming infected or stimulate the production of viruses sheltered by latently infected macrophages once monocytes enter tissues. Furthermore, such MDM repopulating tissues and acquiring self-renewal capacities^47^ could in turn be infected and become HIV reservoirs locally. This could explain how the latently infected pool of macrophages in mucosal tissues is long-lasting and persists despite cART.

In this latter context, the production of endogenous inflammatory factors by monocytes such as alarmins can engage TLRs and participate in endogenous “sterile” inflammation processes^37^. This would in turn promote a local tissue autocrine feedback loop favoring low-level of NF-kB-mediated HIV production despite systemic viral suppression. Such a scenario is in agreement with the presence of whole viral particles in VCCs that we observed in vivo in reservoir macrophages in cART-suppressed patient tissues^12^. Unlike T cells, macrophages produce viral particles that mainly bud into and are stored in VCCs^16^. VCCs may thus contain incoming as well as de novo produced viruses. In the present model, latently infected M4-macrophages containing VCCs stop producing p24 to the extracellular milieu several days post infection. suggesting that the source of virus detected in VCCs is likely not a recently endocytosed virus. However, during the phase of active production of virions by infected macrophages, viruses could not only bud into VCCs^16^, but could be also endocytosed and stored in VCCs, a feature requiring further investigations for which the M4 model is particularly useful.

The presence of virus in VCCs we observed further indicates that these infected tissue macrophages form a low-level productive reservoir, as suggested by others^49^. Maintenance of such low-level replication in HIV-1 reservoirs, i.e., with sustained low-level viral production despite suppressive antiretroviral therapy, would involve a perennial inflammatory stimulus such as those observed in non-resolving inflammation, a chronic condition regulated by macrophages^50,51^.

Produced largely by neutrophils and macrophages, S100A8 forms a heterodimer with the S100 calcium-binding protein A9 (S100A9 or MRP14), and this S100A8/S100A9 complex functions as pro-inflammatory damage-associated molecular patterns (DAMP)^37,38,52^. S100A8 is however the functional unit of the heterodimer able to engage TLR4^38^ that in turn activates NF-κB^53^. This mechanism of action converges with HIV-1 reactivation: indeed, S100A8 activates HIV-1 transcription in latently infected macrophages^54^ and in CD4^+^ T lymphocytes via the NF-κB axis^39^. Therefore, S100A8 could act similarly to the exogenous bacterial component LPS by activating TLR4 signaling, although less strongly and without affecting cell oxygen consumption and mitochondrial respiration. S100A8 might thus be an inflammatory endogenous tissue factor able to stimulate the viral reservoir formed in latently infected macrophages to resume viral production.

Of note, S100A8 systemic levels are increased in HIV-1 patients as well as in other inflammatory diseases and viral infections^38,54–56^. Furthermore, S100A8 levels rise also in genital mucosal secretions from HIV-1-infected patients where it can reactivate HIV-1 replication in latently infected cells^54^. However, in the context of antiretroviral therapy, we found that total S100A8 measured as heterodimer with S100A9 is downregulated in the urethral mucosa of cART-suppressed HIV-1-infected individuals compared with healthy donors. This decrease could be due to a decrease in the mucosa of cART-treated HIV-infected individuals in the frequency of tissue neutrophils that is the most abundant source of S100A8/S100A9 in tissues^38^. Therefore, the specific upregulation of this alarmin in M4-macrophages we observed in cART-suppressed HIV-1-infected individuals compared with healthy donors indicates that S100A8-induced HIV-1 reactivation in macrophage reservoirs likely occurs at a local level under mechanisms remaining to be elucidated. One can propose that S100A8, produced locally by M4-macrophages in a short time window prior to the formation of heterotetramers with S100A9, binds immediately after secretion and in an autocrine/paracrine manner to TLR4 at the M4-macrophage reservoir surface. This interaction would secure a regulatory mechanism for controlling the conversion from HIV-1 deep latency observed in CD4^+^ T cells^57^ toward HIV active transcription and storage of virions in VCCs^12,16^ that we describe here.

One of the mechanisms regulating HIV-1 latency could be metabolic reprogramming, a process central in macrophage pro-reparative/pro-inflammatory plasticity and in response to pathogens^58,59^. Upon pattern recognition receptors that engage DAMPs like S100A8 or pathogen-associated molecular patterns like LPS, macrophages rewire their transcriptional program to produce inflammatory factors and metabolic mediators responsible for the modification of macrophage metabolism for aerobic glycolysis, simultaneously^59^. LPS and S100A8 reverse HIV-1 latency in mucosal macrophage reservoirs, both factors inducing an inflammatory reaction and a glycolytic activity. This property could be used for the elimination of HIV-1 reservoirs^60^. Drugs could be developed to target the glycolytic metabolic pathways, offering an alternative, yet unexplored, for shock-and-kill or block-and-lock strategies. The advantage of such an approach is that macrophages can be “trained” upon recognition of inflammatory stimuli, rewiring their metabolism for days or months, as described in other infectious diseases^61^. Such a long-lasting glycolytic stimulus would keep macrophage reservoirs producing virus until their recognition and elimination by CD8^+^ cytotoxic T lymphocytes-mediated killing, to which macrophages are resistant when acutely infected^62^. Pharmacological inhibition of glycolysis is known to profoundly interfere with the physiology of HIV-1 latently infected cells and HIV-1 reactivation^24,25^. Therefore, a prolonged shift in glycolytic pathways toward different metabolic profiles could prevent macrophage reservoirs from producing viruses, in turn blocking a potential low-level replication of persistent HIV-1 in tissues.

Such blockade of glycolysis could also be exploited to eliminate the HIV-1 reservoir in CD4^+^T cells. Indeed, a glycolytic environment is required to carry out HIV-1 reverse transcription, and inhibition of glycolysis blocks HIV-1 replication, as recently demonstrated^25^. Altogether, the metabolism of cells targeted by HIV-1, either CD4^+^T cells or macrophages, emerges as an important common determinant of HIV-1 infection, even if regulated by different mechanisms.

Overall, our results reveal that a specific macrophage subset, the inflammatory M4 one, forms a viral mucosal HIV-1 reservoir and promotes viral persistence that is regulated by the endogenously expressed S100A8, which would mediate sustained low-level HIV-1 production in the male genital tract. Such novel HIV-1 reservoir in M4-macrophages adds to the blood T-cell reservoir and now needs also to be targeted by eradication strategies. The requirement for glycolysis in HIV-1 reservoir reactivation reveals a stage of vulnerability that can be exploited in future HIV-1 cure strategies.

## Methods

### Ethical statement

The study was conducted under local ethical regulation after approval by the local ethical committee (Comité de Protection des Personnes, Île de France XI; approval number 11 016). Human samples were obtained after written informed consent from all study participants.

### Urethral mucosa tissue cell suspensions and sorting of monocytic/macrophagic urethral cells

Penile tissues were obtained from individuals undergoing elective gender reassignment surgery at the Saint Louis Hospital in Paris, France. These included healthy individuals and also HIV-infected ones (median age of 43 years old [CI: 37–49], infected with HIV-1 for a median duration of 11.5 years [CI: 4–17]) under suppressive cART for a median period of 9.5 years [4–12.9] with undetectable plasma viral loads (<40 RNA copies/mL) for at least 12 months before surgery (Table 1). Immediately following surgery, the penile tissues were preserved in phosphate-buffered saline (PBS) supplemented with 100 U/mL penicillin and 100 μg/mL streptomycin (Gibco, ThermoFisher Scientific) and transported on ice to a biosecurity level 2+ facility. Tissues were processed to obtain a tissue cell suspension of urethral mucosal cells as described^17^. Viability of tissue macrophages after tissue processing was evaluated by DRAQ7 staining (BD biosciences) following the manufacturer’s instructions, before and after removal of dead cells by magnetic isolation (dead cell removal kit, Miltenyi Biotec GmbH). Accordingly, viable macrophages corresponded to >90% (before) and >95% (after dead cell removal) of cells among the population of total tissue macrophages (CD68^+^) in the cell suspension (Supplementary Fig. 3a).

The CD14^+^CD16^+^ cell population was sorted from total tissue cell suspensions using magnetic microbeads (Miltenyi Biotec GmbH) following the manufacturer’s instructions. A mean percentage of 58% [CI: 49.12–66.8] cells in the CD14^+^CD16^+^ selected urethral mucosal cell population corresponds to CD68^+^ macrophages with less than 1% corresponding to CD3^+^ T cells, as quantified by flow cytometry. Potential CD3^+^ cell contaminants were thoroughly washed out from the plates before ex vivo macrophage cultivation, infection, and latency reversal experiments.

### Ex vivo cultivation of urethral macrophages

The total CD14^+^CD16^+^ urethral mucosal cell population was cultivated in flat-bottom well plates (5 × 10^3^ cells per mm^2^ of well area) to which mainly macrophages remained attached after overnight incubation at 37 °C 5% CO_2_ and non-adherent or less adherent cells were thoroughly washed out. The remaining attached tissue macrophages were then cultivated in RPMI 1640 culture medium supplemented with 10% fetal calf serum (FCS) and employed for in vitro HIV infection or HIV latency reversal assays.

### Monocyte isolation and macrophage culture

CD14^+^ cells (monocytes) peripheral blood mononuclear cells obtained by negative selection (STEMCELL Technologies Inc.) according to the manufacturer’s instruction were differentiated in vitro into macrophages. MDM subtypes were obtained using the following regimens in RPMI 1640 with 10% FCS: (i) for M1-macrophages: 6 days of 50 ng/mL GM-CSF (R&D Systems) followed by 20 ng/mL IFN-γ (R&D Systems), and 1 μg/mL LPS (from *Salmonella enterica* serotype typhimurium, Sigma-Aldrich) for additional 2 days; (ii) for M2a-macrophages: 6 days of 25 ng/mL macrophage colony-stimulating factor (M-CSF, R&D Systems) followed by 20 ng/mL IL-4 (R&D Systems) and 20 ng/mL IL-13 (R&D Systems) cytokines for additional 2 days^63^; or 3 (iii) for M4-macrophages: 30 μg/mL CXCL4/PF4 (Chromatec GmbH) for 6 days^26,31^. Non-polarized MDM were produced by cultivation in a complete medium supplemented with 50 ng/mL GM-CSF and 25 ng/mL M-CSF for 6 days.

### In vitro and ex vivo HIV infection

In vitro and ex vivo infection experiments were performed using MDM from healthy donors or urethral tissue macrophages respectively, cultivated in the presence of 20 ng/mL [p24-Gag] R5-tropic HIV-1 primary isolate (93BR029, NIH AIDS Reagents program) for 2 h. Virus was then removed by thorough washes and a fresh complete medium was added to macrophage cultures. Zidovudine (AZT) (NIH AIDS Reagent Program) was added at 10 μM to ex vivo-infected tissue macrophages as a negative control when quantifying productive infection. Viral production in the days following infection was quantified in culture supernatants by ELISA for HIV-1 p24-Gag (p24 Innotest HIV-1 ELISA, InGen, Diaxonhit group, France) for up to 60 days. We defined that viral latency was reached when viral detection in the macrophage culture supernatants become undetectable by the p24 ELISA employed as we described^6^. Integrated HIV DNA was assessed by Alu-gag nested PCR as described^12^.

### Latency reversal assays

Latently infected macrophages, either infected in vitro or selected from urethral tissue of cART-suppressed HIV-infected individuals, were treated with 1 μg/mL of bacterial LPS which reactivates viral production^6,9,11,12^, and with 5–20 μg/mL of purified S100 calcium-binding protein A8 (S100A8)^37^. LPS or S100A8 were added to macrophage cultures for 48 h to reactivate viral production that was then measured in culture supernatants.

In latently infected M4-MDM, p24-Gag ELISA was performed directly on culture supernatants after 48 h of latency reversal with LPS or S100A8 (5–20 μg/mL). Cell fractions were processed for HIV-1 LTR RT-qPCR as described^64^. Reversal of HIV latency was carried out in the presence of 10 μM Zidovudine (AZT) in some experiments.

In tissue macrophages obtained from healthy donors and latently infected in vitro, p24-Gag ELISA was performed in the days following infection until HIV-1 production becomes undetectable by this method. Next, the tissue macrophages were reactivated with LPS or S100A8 (10 μg/mL) for 48 h, and the macrophages were processed for HIV-1 LTR RT-qPCR as described^64^. To take into account the variability in the acute HIV-1 infection level of tissue macrophages in different donor samples, the amount of virus produced upon reactivation was expressed by the ratio between the number of HIV-1 LTR copies found in reactivated macrophages and the maximal cumulative production of p24-Gag detected by ELISA before the latency.

In tissue macrophage reservoirs obtained from cART-suppressed HIV-1-infected individuals and reactivated ex vivo with LPS or S100A8 (10 μg/mL), supernatants were collected 48 h after reactivation. To amplify the small amounts of replication-competent virus produced in this case, supernatants were added to CD4^+^ T-cell lymphoblasts in a viral outgrowth assay, as described below.

### Low-level infectious HIV production assessed by viral outgrowth assays

Due to the limited amount of latently infected macrophages generated in vitro^24^ or obtained in vivo^12^ and also as the amount of virus released into culture supernatant from latently infected macrophages reactivated in vitro is below the limit of detection for most conventional techniques, it was necessary to amplify the reactivated HIV by outgrowth assays using CD4^+^ T-cell lymphoblasts^12,65^.

Accordingly, the production of replication-competent virus following HIV latency reversal of infected macrophages was quantified by an adapted viral outgrowth assay performed as follows: after 2 days of latency reversal, supernatants of infected macrophages were incubated with CD4^+^ T-cell lymphoblasts from healthy donors produced as described^65^ for 7 days in RPMI 1640 with 10% FCS supplemented with 10 U/mL IL-2 (Sigma-Aldrich). Lymphoblasts were centrifuged at 300 g, 10 min, and resuspended in a fresh complete medium containing 10 U/mL IL-2 for additional 7 days. Then, lymphoblasts were centrifuged again and cell pellets were processed for RT-qPCR of HIV-1 LTR as described^64^.

### Multiparametric flow cytometry

Multiparametric flow cytometry was performed as described^12^ with simplifications in the gating strategy: our previous experience with urethral tissues^12^ allowed us to identify dead cells in FSC/SSC dot plots of tissue cells suspension, gating viable cells (Supplementary Fig. 3a, b). Doublets were excluded in an FSC-A/FSC-H dot plot and macrophages were gated in a CD3/CD68 dot plot as CD68^+^CD3^neg^ cell population (Supplementary Fig. 3b, c). The antibodies used in this study were designed and validated for flow cytometry and are disclosed in Supplementary Table Data 1. Immunofluorescence of labeled tissue cell suspensions was acquired on an LSR II cytometer (BD Biosciences). Fluorescence specificity and compensation were established in the BD CellQuest (BD Biosciences) data acquisition software using BD CompBead Compensation Particles (BD Biosciences) for each fluorescence-coupled antibody used in the panel.

Flow cytometry data were analyzed with FlowJo software (FlowJo version X 10.0.7r2; FlowJo, LCC). UMAPs were generated by concatenation of CD68^+^ cell population of four cART-treated HIV-infected individuals followed by FlowJo UMAP plugin (S100A8, MMP7, CD206, CD163, IL-1R, and IL-4R markers used). Clustering analysis on UMAP was performed by FlowSom plugin to define the different macrophage populations.

The correlation between the expression of different markers comparing macrophages from different origins (derived in vitro or obtained from urethral tissues) was performed by ClustVis software^66^ using the mean percentage of macrophages expressing these markers in a total population of macrophages. The correlation analysis is shown as a heatmap generated using the following parameters: correlation as clustering distance and average as a method for clustering between different macrophage samples; tree ordering set as tightest cluster first.

### Fluorescence in situ hybridization coupled to flow cytometry (FISH-flow)

The detection of HIV-infected cells in the tissue cell suspensions was performed by immunostaining of viral protein p24-Gag (anti-p24 clone KC57, FITC), combined with fluorescence in situ hybridization using mRNA Cy5-tagged probes covering *gag*, *pol*, and *env* HIV genes, both labeling being quantified at the single-cell level by flow cytometry, a technique described as FISH-flow^67^. Probes were designed by Stellaris Probe Designer program ((https://www.biosearchtech.com/support/tools/design-software/stellaris-probe-designer) as we described^34^ and the probe sets are detailed in Supplementary Table Data 1. FISH-flow recently succeeded in detecting the very low amount of latent HIV-1 reservoir cells in cART-treated patients, which comprises around 1 infected cell per million CD4^+^ T cells^35,67,68^. The technique was performed here as described^64^ and was employed in the context of multiparametric flow cytometry using a similar gating strategy as described above (Supplementary Fig. 4c).

### Quantification of PF4 expression in tissues

Paraformaldehyde-fixed, paraffin-embedded urethral tissue pieces were sectioned (4 μm), deparaffinized in xylene and graded alcohol solutions, treated with Hydrogen Peroxide solution (Advanced Cell Diagnostics, Inc.) for 10 min, heated in boiling RNAscope target retrieval buffer (Advanced Cell Diagnostics, Inc.) for 30 min as recommended by the manufacturer for antigen retrieval, incubated with RNAscope Protease Plus (Advanced Cell Diagnostics, Inc.) for 20 min at 40 °C, and washed in distilled water. Sections were then blocked for nonspecific staining in a blocking solution (1% w/v BSA, 1% v/v horse serum, 5% v/v human serum, 1% g/v gelatin, and 50 mM EDTA) for 1 h at room temperature. Next, sections were labeled with 10 μg/mL CXCL4/PF4 (Chromatec, a-PF4-h1, IgG2b) and 20 μg/mL CD68 (Novus, NB100-683, IgG1) antibody overnight at 4 °C. After washing in blocking solution, sections were incubated with 1:200 v/v Cy3-conjugated-anti-mouse-IgG2b (Jackson ImmunoResearch, 115-165-207) and 1:200 v/v Cy5-conjugated-IgG1 (Abcam, ab136127) secondary antibodies for 2 h at room temperature. Finally, sections were washed, counterstained with 4′,6-diamidine-2′-phenylindole dihydrochloride (DAPI), and mounted with a mounting medium (ibidi, 50001). Immunofluorescence was visualized using a Spinning disk unit (Ixplore, Olympus) and analyzed using FUJI-image J software^69^. Three scans were taken from each section at ×30 magnification. CXCL4/PF4 positive pixels and area per unit area were calculated as described^70^.

### Morphological analyses of VCCs

Confocal microscopy after immunostaining was performed as described^6,12^ using anti-CD68 (R&D systems, 10 μg/mL) and anti-p24-Gag antibodies (EVA365 and EVA366, 1:10 v/v each, NIBSC, Center for AIDS Reagents). Acquired z-stacks were reconstructed into three-dimensional image projections in Imaris software (version 9.0.2, Oxford Instruments plc, UK) and visualized using MIP filters. Imaris creates isosurface objects by filtering and segmenting the original dataset, attributing dimensional data to these isosurfaces such as volume (μm^3^)^71^. The attribution of isosurface objects based on p24-Gag immunostaining in infected macrophages allowed for retrieving volumetric measures of the cluster of p24-Gag signal displayed by these infected cells, corresponding to VCC^12,16^. The sum of the volumes measured by all VCCs detected in one cell represents the VCC volume per cell referred in this study, as described^72^.

### Morphological analyses following in situ hybridization

Urethral macrophages were cultivated ex vivo in 8-well detachable chamber glass slides (ThermoFisher Scientific) for HIV RNA in situ hybridization as described^6,73^, using the RNAscope Multiplex Fluorescent V2 Assay kit (Advanced Cell Diagnostics, Inc.) following the manufacturer’s instructions including treating samples with 30 min of boiling Target Retrieval Reagent and 10 min at 40 °C of Protease Plus before hybridization and amplification steps of the protocol.

For combined detection of HIV RNA and p24-Gag immunostaining, after in situ hybridization procedures, samples were treated for 1 h with a blocking buffer containing 50 mM EDTA, 0.45% cold water fish gelatin (Sigma-Aldrich), 10 mg/mL BSA fraction V (Sigma-Aldrich), 1:100 v/v horse serum and 1:20 v/v human serum in water. Next, samples were incubated overnight at 4 °C with 1:1000 v/v of anti-HIV-1 p24-Gag monoclonal antibody (NIH AIDS Reagent Program, catalog number 6457) diluted in a blocking buffer. Samples were then incubated with anti-mouse IgG1 secondary antibody coupled to AlexaFluor 488 1:2000 v/v diluted in blocking buffer, washed in PBS, and incubated with 10 μM DAPI for 15 min before coverslips were mounted on slides with anti-fading medium suitable for confocal laser scanning microscopy.

The same protocol was applied to FISH and immunostaining of CD68 and S100A8, using for detection of these markers the following primary and secondary antibodies: anti-human CD68 (R&D MAB20401) and anti-human S100A8/S100A9 heterodimer (R&D MAB45701) both at 10 μg/mL, and anti-mouse IgG2b coupled to AlexaFluor 488 (Jackson 115-545-207) or anti-rabbit IgG coupled to Cy5 (Jackson 711-177-003) both at 1:200 v/v.

The complete list of antibodies used in this study is found in Supplementary Table Data 2.

### Evaluation of macrophage glycolytic metabolism

ECAR and OCR were measured using the XFe96 Analyzer (Agilent Technologies) as described by the manufacturer.

To compare the metabolism of M4- and M2-macrophages, macrophages were preincubated with Seahorse XF DMEM medium pH 7.4 (Agilent) supplemented with 2 mM glutamine (Glucose-free Seahorse medium). The instrument was programmed to sequentially inject compounds into wells of the Seahorse culture plates in the following order: glucose (11 mM final well concentration), etomoxir (Sigma-Aldrich) (100 μM final well concentration), 2-DG (Sigma-Aldrich) (10 mM final well concentration), and electron transport chain inhibitors antimycin A and rotenone (AM/rot)(Agilent) (10 μg/mL each final well concentration). Compounds were diluted in Glucose-free Seahorse medium.

To compare changes in M4-macrophage metabolism upon S100A8 or LPS treatment, macrophages were preincubated with Seahorse XF DMEM medium pH 7.4 (Agilent) supplemented with 2 mM glutamine and 11 mM glucose (complete Seahorse medium). The instrument was programmed to sequentially inject: first, the Seahorse medium alone, LPS (1 μg/mL final well concentration) or S100A8 (10 μg/mL final well concentration) in Seahorse medium; second, etomoxir (100 μM final well concentration); third 2-DG (10 mM final well concentration) and fourth, AM/rot (each 10 μg/mL final well concentration). Compounds were diluted in a complete Seahorse medium.

Data obtained were first analyzed by Agilent Seahorse Wave Desktop software (Agilent Technologies) and normalized to cells numbers per well (quantified by microscopic automatic counting algorithms using Imaris software and DAPI fluorescent staining) and to baseline ECAR/OCR levels (last measure before the first injection). ECAR/OCR data is therefore shown as the mean percentage after baseline normalization (baseline levels defined as 100%). The glycolytic activity refers to a subtraction of the ECAR peak level after the first injection and the minimum ECAR level after 2-DG injection.

Energy maps were generated by plotting these ECAR peak levels against corresponding OCR measures acquired at the same time point. ECAR/OCR baseline levels (last measure before the first injection) were normalized to correspond to 100. Arrows indicate the increase in either ECAR or OCR levels between baseline measures (before the first injection) and the ECAR peak (after the first injection).

### Cytokines/chemokine quantification

Urethral mucosa tissues were processed as described^74^ to obtain tissue lysates. The cytokines/chemokines CXCL4/PF4, IL-13, M-CSF, MCP-1/CCL2, TRAIL, RANTES/CCL5, MIP-1α/CCL3, GM-CSF, IL-4, MIP-3α/CCL20, and MCP-4/CCL13 were quantified using the Luminex technology (LXSAHM, R&D) on a Bio-Plex 200 (Bio-Rad) according to the manufacturer’s recommendations. IFN-γ was quantified using the Human IFN-γ ELISA Development Kit (Mabtech), according to the manufacturer’s instructions. The lower limit of quantification of each cytokine/chemokine is 2 pg/mL (CCL13/MCP-4 and IFNγ), 3 pg/mL (CCL20/MIP-3α), 4 pg/mL (GM-CSF), 7 pg/mL (CCL5/RANTES), 10 pg/mL (TRAIL and CCL2/MCP-1), 16 pg/mL (IL-4), 100 pg/mL (CXCL4/PF4 and CCL3/MIP-1a), and 140 pg/mL (IL-13 and M-CSF). Correlograms were generated in R using *cor*, *cor_test_mat*, and *corrplot* functions.

S100A8/S100A8 heterodimer was measured by Quantikine ELISA kit (DS8900, R&D) according to the manufacturer’s recommendations.

Cytokine levels were normalized by the protein amount in tissue lysates (mg/mL), quantified by NanoDrop spectrophotometer (ThermoFisher Scientific) using the program Protein A280.

### Statistical analysis

The results are represented as means with respective standard errors or boxplots. Boxplots represent minima and maxima as lower and upper whiskers, median as a center of the box, and the interquartile range Q1 and Q3 as lower and upper bounds. Statistical tests were performed by SPSS software (IBM), considering normal (parametric tests, Student’s *t*-test or ANOVA with post hoc pairwise comparisons) or non-normal distributions (non-parametric tests, Mann–Whitney or Kruskal–Wallis), and two-sided tests. Significant differences were indicated by asterisks considering *p* values below 0.05. For cytokine correlograms, *cor_test_mat* function was employed with Benjamini & Hochberg adjustment.

### Reporting summary

Further information on research design is available in the Nature Research Reporting Summary linked to this article.

## Supplementary information

Supplementary Information

Description of Additional Supplementary Files

Supplementary Dataset 1

Supplementary Dataset 2

Reporting Summary

## Data Availability

All data associated with this study are in the paper or supplementary materials. Source Data are provided with this paper.

## Source data

Source Data
