# Supplementary material for: S100A8-mediated metabolic adaptation controls HIV-1 persistence in macrophages in vivo
Source: Nat Commun. 2022 Oct 11;13:5956. doi: 10.1038/s41467-022-33401-x (PMC9553955; doi:10.1038/s41467-022-33401-x)
Supplement: Supplementary file 1 — Supplementary Information [file 41467_2022_33401_MOESM1_ESM.docx]

**Real et al.**

S100A8-mediated metabolic adaptation controls HIV-1 persistence in macrophages *in vivo*

**Supplementary Information**

**Supplementary Tables**

**Supplementary Table Data** **1. List of HIV-1 mRNA probe sequences used for FISH-flow.** List of 48 mRNA probe sequences used for FISH-flow analysis of macrophage reservoirs, comprising *gag*, *pol* and *env-nef* mRNA (HIV-1 vector pNL4-3; GenBank AF324493.2). Probes were designed using the Stellaris Probe Designer program available at http://www.singlemoleculefish.com.


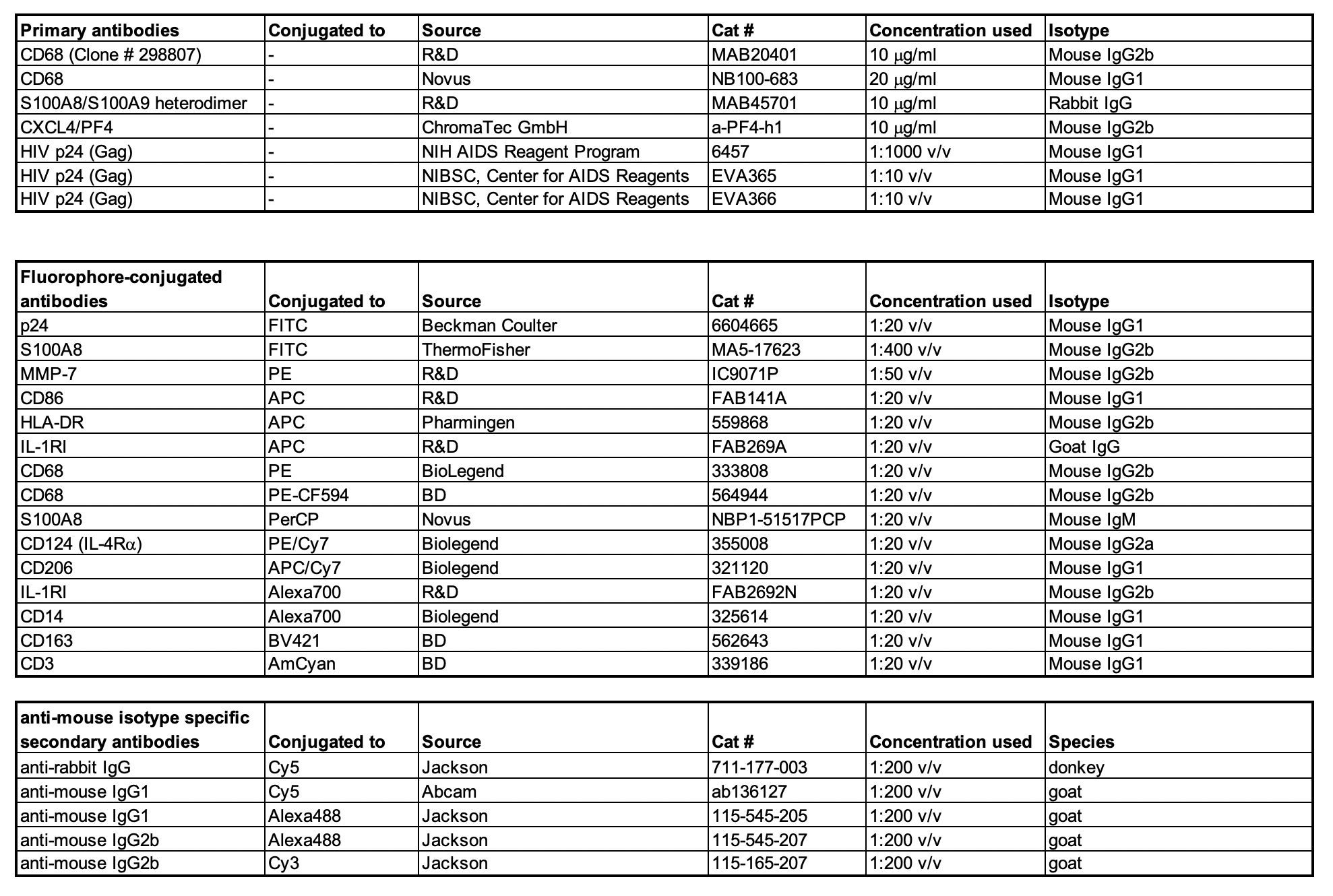


**Supplementary Table Data 2. List of antibodies used in this study.**

**Supplementary Figures**


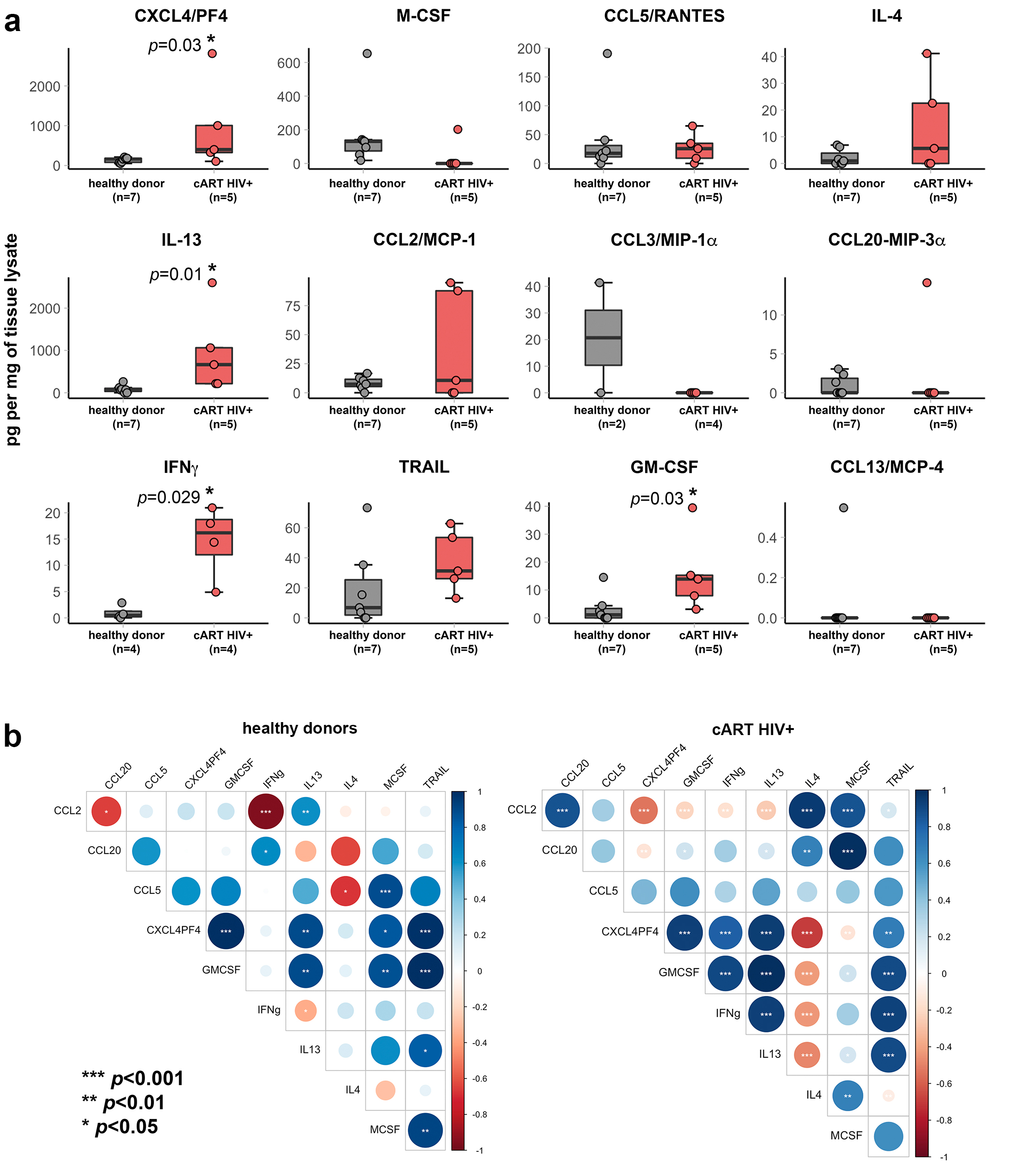


**Supplementary Figure 1. Supplementary information for cytokine multiplex data.**

(a) Full cytokine profile of urethral tissue extracts of healthy donors (grey) and cART-suppressed HIV-infected (cART HIV^+^) individuals (red). The number of individual samples (n) included in the analysis is shown per group and cytokine tested. Mann-Whitney test performed for pairwise comparisons between the two groups of individuals and per cytokine. Asterisks indicate statistically significant differences at *p*<0.05. Source data are provided as a Source Data file.

(b) Correlation map of cytokine levels quantified in urethral mucosa of healthy donors (left, n=7 except for IFN-γ where n=4) and cART HIV^+^ individuals (right, n=5 except for IFN-γ where n=4). Positive correlations have blue colors. Negative correlations have red colors. The intensity of the color and the size of circles both follow the level of correlation indicated in the scale bars close to the maps. Asterisks indicate statistical significance at *p*<0.001 (***), *p*<0.01 (**) and *p*<0.05 (*) after Benjamini & Hochberg (BH) adjustment for multiple comparisons. Source data are provided as a Source Data file.


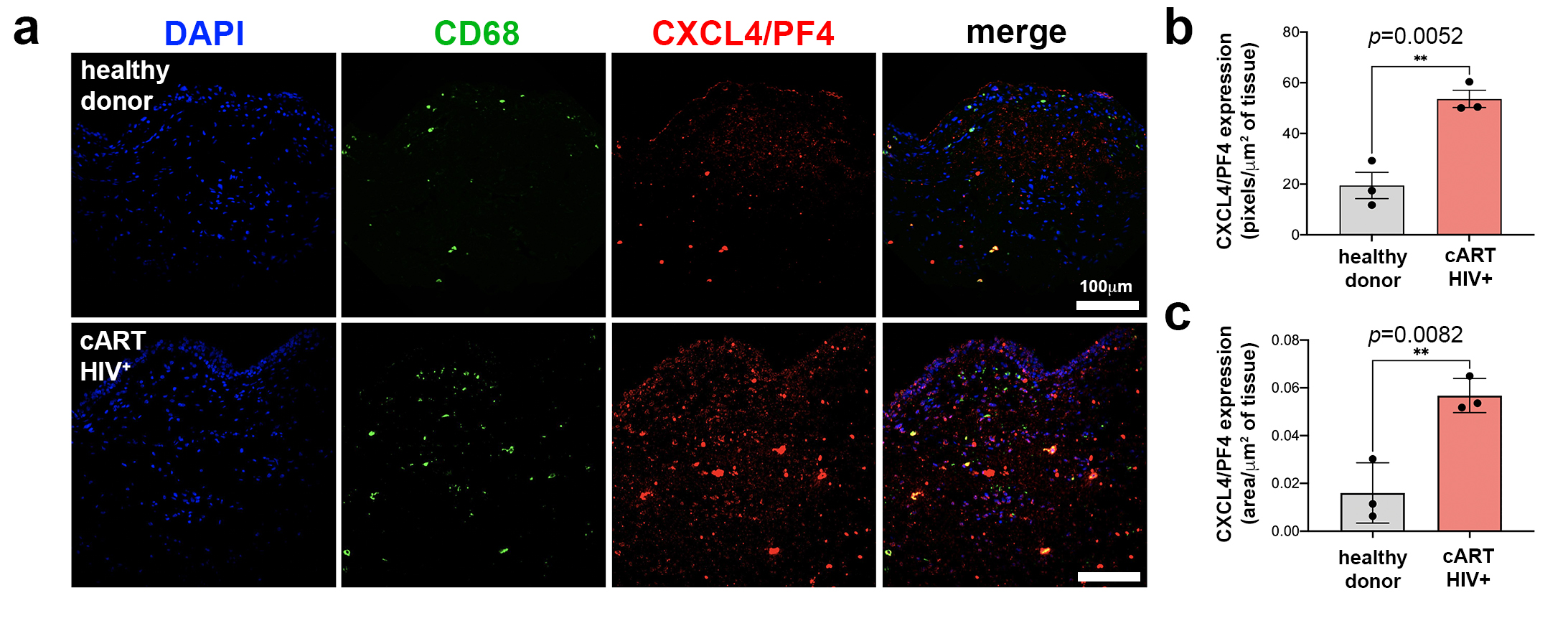


**Supplementary Figure 2. CXCL4/PF4 expression in the urethral mucosa.**

(a) Immunofluorescence staining of CXCL4/PF4 (red) and CD68 (green) in human urethra tissue sections. Nuclear counterstain is shown in blue. Images are representative of sections from healthy donors (n=3) and cART HIV^+^ donors (n=3). Bar= 100 μm.

(b-c) Quantification of CXCL4/PF4 staining signal, calculated as the CXCL4/PF4 pixel per μm^2^ (b) and CXCL4/PF4 area per μm^2^ (c) acquired from 3 different microscopic fields per donor scanned at x30 magnification. Asterisk indicate statistical significance at *p*<0.05 (Student’s *t*-test). Source data are provided as a Source Data file.

**
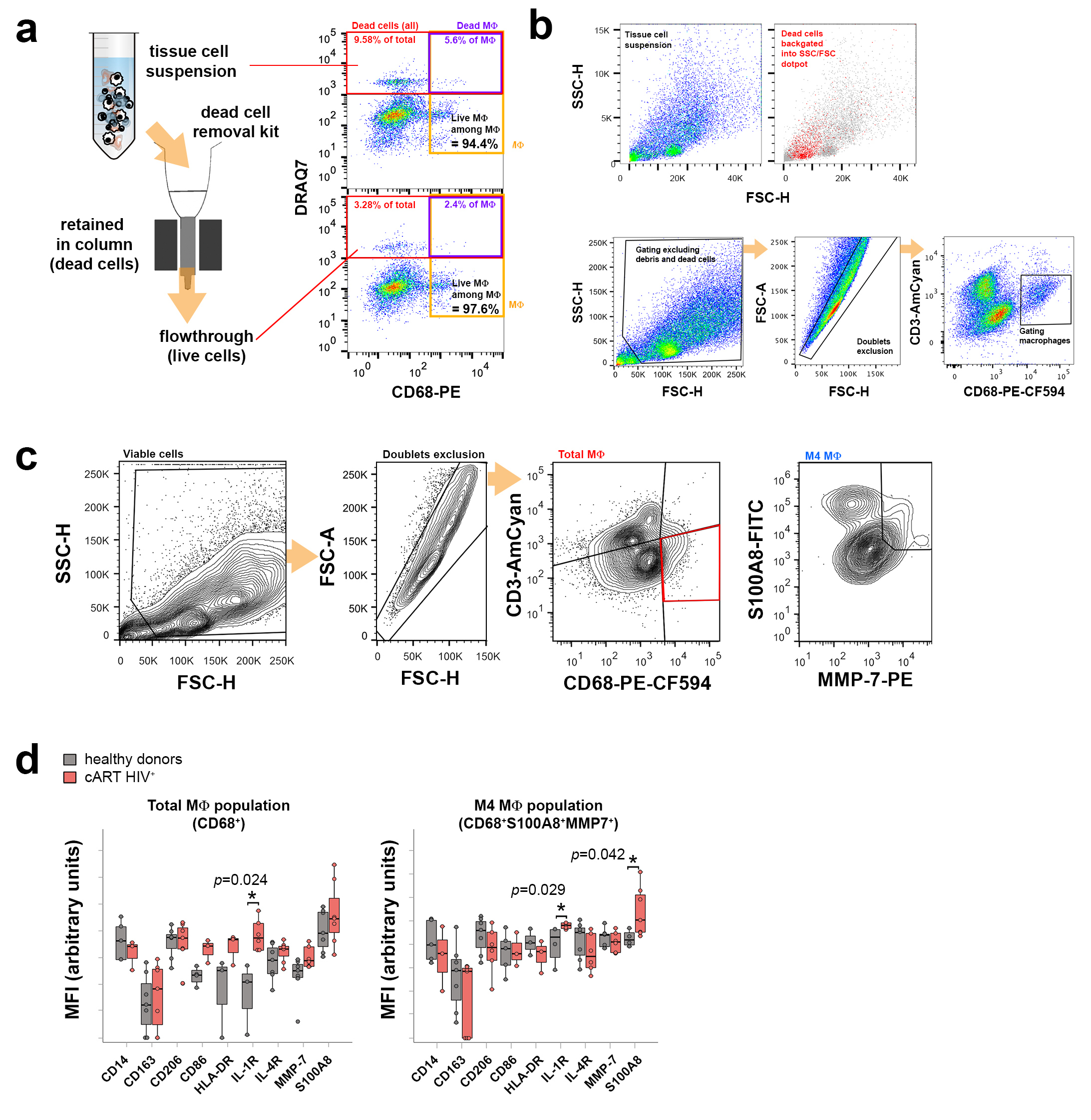
**

**Supplementary Figure 3. Supplementary information for Flow cytometry**

(a) Experimental strategy to quantify viability of urethral macrophages after tissue processing by DRAQ7 staining and magnetic exclusion of dead cells.

(b) Gating strategy to quantify urethral tissue macrophages: exclusion of dead cells based on FSC/SSC dot plots (validated by DRAQ7 staining) followed by doublets exclusion in FSC-A/FSC-H dot plots and gating of CD68^+^CD3^neg^ cell population.

(c) Gating strategy to assess M4-macrophages in urethral tissue cell suspensions.

(d) Mean fluorescence intensity (Geomean) of macrophage polarization markers displayed by total MΦ among the tissue cell suspension (Total MΦ population, left graph) and M4-macrophages (M4-macrophages population, right graph) in healthy donors (grey, n=7) and cART-suppressed HIV-infected individuals (red, n=7). Mann-Whitney test between healthy donor and cART HIV^+^ group: asterisks indicate *p*<0.05. Source data are provided as a Source Data file.

**
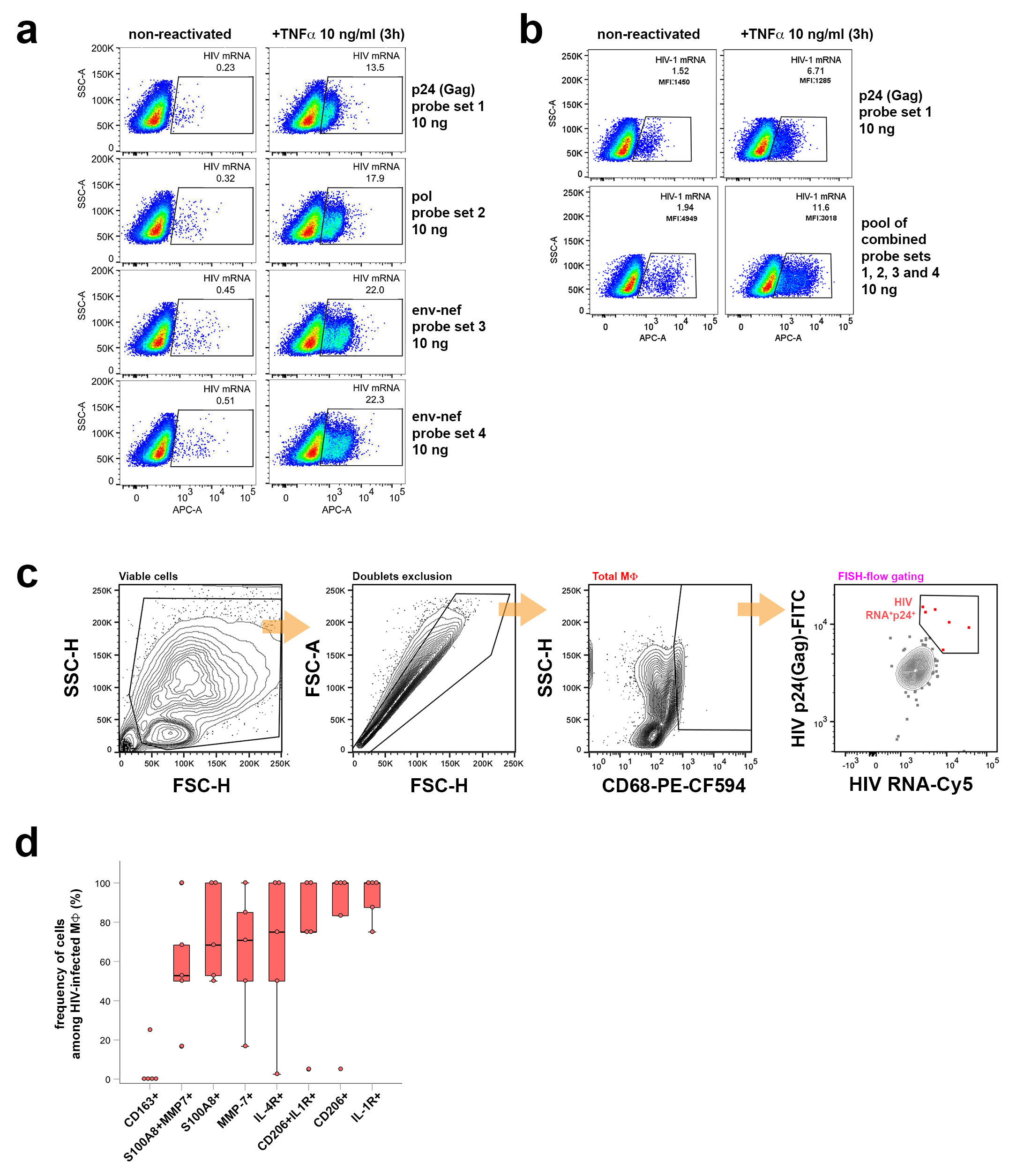
**

**Supplementary Figure 4. Supplementary information for FISH-flow data.**

(a-b) Validation of FISH-flow probes in a model of reactivation of viral production. (a) Flow cytometry gating of HIV RNA^+^ events from OM-10.1 cells, which contain integrated provirus and express constitutively low amounts of HIV-1, treated or not with 10 ng/ml TNF-α for 3 hours and processed for FISH-flow using four different probe sets (targeting *gag*, *pol* and *env-nef* mRNA individually). (b) TNF-α-mediated reactivation of viral production of OM-10.1 cells followed by FISH-flow using a single probe set (targeting *gag* mRNA, upper) as compared with the four probe sets together targeting *gag*, *pol* and *env-nef* mRNA (lower).

(c) Gating strategy to assess HIV RNA^+^/p24-Gag^+^ MΦ in urethral tissue cell suspensions.

(d) Frequency of macrophage markers displayed by HIV-infected macrophages (percentage among HIV RNA^+^ CD68^+^ MΦ). Data were obtained from five different cART HIV-infected individuals. Source data are provided as a Source Data file.


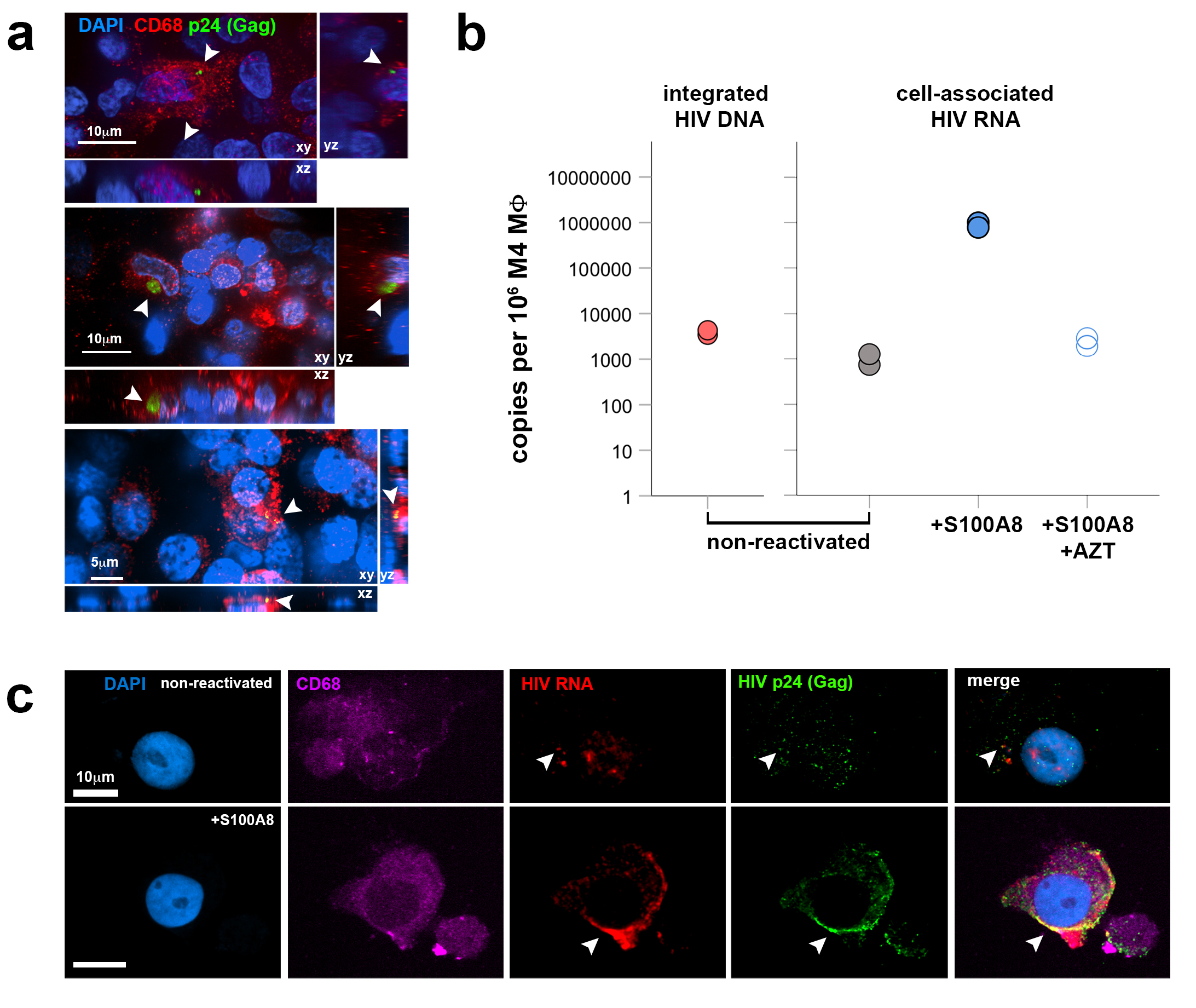


**Supplementary Figure 5. Supplementary information for *in vivo-* and *in vitro*-infected macrophages.**

(a) Additional confocal microscopy images of macrophage reservoirs from two cART-suppressed HIV-1 individuals. Infected tissue macrophages obtained from cART-suppressed HIV-infected individuals immunostained for p24-Gag (green) and CD68 (red). Nuclei were stained with DAPI. Images are shown as xy projections (main figures) and xz (bottom)/yz projections (right). Bar= 5 or 10 μm.

(b) Integrated HIV DNA and HIV RNA quantified in HIV-1-latently infected M4-macrophages. Integrated HIV DNA was detected by Alu-gag nested PCR in non-reactivated latently infected M4. HIV RNA was detected by RT-qPCR in non-reactivated and S100A8-reactivated latently infected M4, treated or not with Zidovudine (AZT). Data were obtained from M4-MDM differentiated from a pool of three healthy donors. Dots represent technical replicates. Source data are provided as a Source Data file.

(c) Tissue macrophage reservoirs isolated from cART-suppressed HIV-infected individual after S100A8-induced latency reversal *ex vivo*, observed by confocal microscopy following HIV RNA *in situ* hybridization coupled to p24-Gag and CD68 immunostaining. Upper row shows non-reactivated tissue macrophage reservoirs with clustered HIV RNA and p24-Gag signals; lower row shows S100A8-reactivated tissue macrophage reservoirs with HIV RNA and p24-Gag signals colocalizing at macrophage membrane. Columns refer to CD68 (magenta), Nuclei (DAPI, blue), HIV RNA (red), p24-Gag (green) staining and merged fluorescence signals. Image is representative of two cART-treated HIV-infected individual samples.


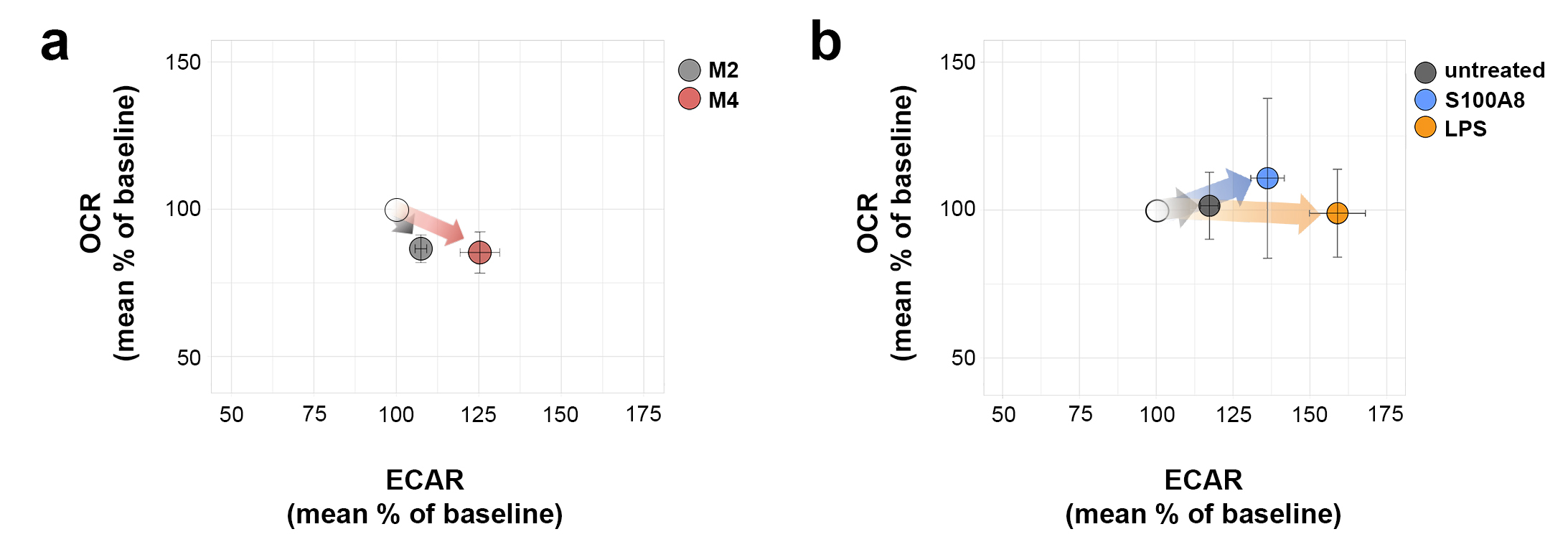


**Supplementary Figure 6. Energy map correlating ECAR and OCR levels.**

(a) ECAR vs OCR levels in M2-MDM or M4-MDM at the peak of the glycolytic activity (maximum ECAR after injection of glucose, corresponding OCR levels at the same time point). Energy maps were generated by plotting ECAR peak levels after glucose injection against corresponding OCR measures acquired at the same time point. Data are presented as mean values +/- SEM and were obtained from four independent experiments. Source data are provided as a Source Data file.

(b) ECAR vs OCR levels in M4-MDM at the peak of glycolytic activity after injection of medium alone, S100A8 or LPS (maximum ECAR after these injections and corresponding OCR levels at the same time point). Energy maps were generated by plotting ECAR peak levels after medium, S100A8 or LPS injection against corresponding OCR measures acquired at the same time point. Data is normalized by baseline ECAR/OCR levels acquired before injections, to a value of 100. Arrows indicate the increase in ECAR/OCR from the baseline for each condition studied. Data are presented as mean values +/- SEM and were obtained from four independent experiments. Source data are provided as a Source Data file.
