# Supplementary material for: S100A8-mediated metabolic adaptation controls HIV-1 persistence in macrophages in vivo
Source: Nat Commun. 2022 Oct 11;13:5956. doi: 10.1038/s41467-022-33401-x (PMC9553955; doi:10.1038/s41467-022-33401-x)
Supplement: Supplementary file 2 — Description of Additional Supplementary Files [file 41467_2022_33401_MOESM2_ESM.pdf]

Real et al.

S100A8-mediated metabolic adaptation controls HIV-1 persistence in macrophages *in vivo*

Supplementary Dataset 1: List of HIV-1 mRNA probe sequences used for FISH-flow.

Supplementary Dataset 2: List of antibodies used in this study.
