# Supplementary material for: S100A8-mediated metabolic adaptation controls HIV-1 persistence in macrophages in vivo
Source: Nat Commun. 2022 Oct 11;13:5956. doi: 10.1038/s41467-022-33401-x (PMC9553955; doi:10.1038/s41467-022-33401-x)
Supplement: Supplementary file 5 — Reporting Summary [file 41467_2022_33401_MOESM5_ESM.pdf]

nature portfolio

Corresponding author(s): Morgane BOMSEL

Last updated by author(s): Aug 23, 2022

Reporting Summary

Nature Portfolio wishes to improve the reproducibility of the work that we publish. This form provides structure for consistency and transparency in reporting. For further information on Nature Portfolio policies, see our Editorial Policies and the Editorial Policy Checklist.

Statistics

For all statistical analyses, confirm that the following items are present in the figure legend, table legend, main text, or Methods section.

n/a	Confirmed	
☐	☒	The exact sample size (n) for each experimental group/condition, given as a discrete number and unit of measurement
☐	☒	A statement on whether measurements were taken from distinct samples or whether the same sample was measured repeatedly
☐	☒	The statistical test(s) used AND whether they are one- or two-sided<br>*Only common tests should be described solely by name; describe more complex techniques in the Methods section.*
☐	☒	A description of all covariates tested
☐	☒	A description of any assumptions or corrections, such as tests of normality and adjustment for multiple comparisons
☐	☒	A full description of the statistical parameters including central tendency (e.g. means) or other basic estimates (e.g. regression coefficient) AND variation (e.g. standard deviation) or associated estimates of uncertainty (e.g. confidence intervals)
☐	☒	For null hypothesis testing, the test statistic (e.g. F, t, r) with confidence intervals, effect sizes, degrees of freedom and P value noted<br>*Give P values as exact values whenever suitable.*
☒	☐	For Bayesian analysis, information on the choice of priors and Markov chain Monte Carlo settings
☒	☐	For hierarchical and complex designs, identification of the appropriate level for tests and full reporting of outcomes
☒	☐	Estimates of effect sizes (e.g. Cohen's d, Pearson's r), indicating how they were calculated

Our web collection on statistics for biologists contains articles on many of the points above.

Software and code

Policy information about availability of computer code

Data collection	All software used are commercially available and details of which software version is stated in the method section. Millipore InCyte and GuavaSuite (GuavaSoft 3.3), BD LSR-II CellQuest, Seahorse acquisition software, LightCycler 480 v1.5
Data analysis	All software used are commercially available and details of which software version is stated in the method section. Imaris 9.0.2, SPSS 22, R 4.1.3 and Rstudio, ClustVis (https://biit.cs.ut.ee/clustvis/), FlowJo 10.0.7r2, Seahorse Wave 2.4.

For manuscripts utilizing custom algorithms or software that are central to the research but not yet described in published literature, software must be made available to editors and reviewers. We strongly encourage code deposition in a community repository (e.g. GitHub). See the Nature Portfolio guidelines for submitting code & software for further information.

Data

Policy information about availability of data

All manuscripts must include a data availability statement. This statement should provide the following information, where applicable:

- Accession codes, unique identifiers, or web links for publicly available datasets
- A description of any restrictions on data availability
- For clinical datasets or third party data, please ensure that the statement adheres to our policy

All data associated with this study are in the paper or supplementary materials. Source data are provided with this paper.

Human research participants

Policy information about studies involving human research participants and Sex and Gender in Research.

Reporting on sex and gender	This study adress the male genital tract and therefore our finding applies to male tissues.
Population characteristics	Clinical data of HIV-1-infected cART-suppressed individuals, from whom penile tissues are obtained and used in the study, are detailed in Table 1
Recruitment	Urethral tissues were obtained consecutively from healthy and HIV-1-infected cART-suppressed individuals undergoing elective gender assignment surgery at the Saint Louis Hospital in Paris. Tissues were excluded from the study if individuals had clinical history of other sexually-transmitted infections in the 6 months prior to surgery
Ethics oversight	local ethical committee (Comité de Protection des Personnes, Île de France XI; approval number 11 016).

Note that full information on the approval of the study protocol must also be provided in the manuscript.

Field-specific reporting

Please select the one below that is the best fit for your research. If you are not sure, read the appropriate sections before making your selection.

☒ Life sciences ☐ Behavioural & social sciences ☐ Ecological, evolutionary & environmental sciences

For a reference copy of the document with all sections, see nature.com/documents/nr-reporting-summary-flat.pdf

Life sciences study design

All studies must disclose on these points even when the disclosure is negative.

Sample size	No sample-size calculations have been preformed in this study. At least n=3 biological triplicates were performed in all experiments unless explicitely indicated
Data exclusions	No data were excluded from the data analysis
Replication	All attempts at replication were successful
Randomization	No experimental groups were allocated in this study
Blinding	Blinding was not performed and is not relevant in this study

Reporting for specific materials, systems and methods

We require information from authors about some types of materials, experimental systems and methods used in many studies. Here, indicate whether each material, system or method listed is relevant to your study. If you are not sure if a list item applies to your research, read the appropriate section before selecting a response.

Materials & experimental systems

n/a	Involved in the study	
☐	☒	Antibodies
☐	☒	Eukaryotic cell lines
☒	☐	Palaeontology and archaeology
☒	☐	Animals and other organisms
☒	☐	Clinical data
☒	☐	Dual use research of concern

Methods

n/a	Involved in the study	
☒	☐	ChIP-seq
☐	☒	Flow cytometry
☒	☐	MRI-based neuroimaging

Antibodies

Antibodies used	All antibodies used are detailled in the method section. They are: CD68 (Clone # 298807, R&D, MAB20401); CD68 (Novus, NB100-683); S100A8/S100A9 heterodimer (R&D, MAB45701); CXCL4/PF4 (ChromaTec GmbH, a-PF4-h1); HIV p24 (Gag)(NIH AIDS Reagent Program, 6457); HIV p24 (Gag)(NIBSC, Center for AIDS Reagents, EVA365); HIV p24 (Gag)(NIBSC, Center for AIDS Reagents, EVA366); p24-FITC (Beckman Coulter, 6604665);

	S100A8-FITC (ThermoFisher, MA5-17623); MMP-7-PE (R&D, IC9071P); CD86-APC (R&D, FAB141A); HLA-DR-APC (Pharmingen, 559868); IL-1RI-APC (R&D, FAB269A); CD68-PE (BioLegend, 333808); CD68-PE-CF594 (BD, 564944); S100A8-PerCP (Novus, NBP1-51517PCP); CD124 (IL-4Ra)-PE/Cy7 (Biolegend, 355008); CD206-APC/Cy7 (Biolegend, 321120); IL-1RI-Alexa700 (R&D, FAB2692N); CD14-Alexa700 (Biolegend, 325614); CD163-BV421(BD, 562643); CD3-AmCyan (BD, 339186); anti-rabbit IgG-Cy5 (Jackson, 711-177-003); anti-mouse IgG1-Cy5 (Abcam, ab136127); anti-mouse IgG1-Alexa488 (Jackson, 115-545-205); anti-mouse IgG2b-Alexa488 (Jackson, 115-545-207); anti-mouse IgG2b-Cy3 (Jackson, 115-165-207)
Validation	All antibodies used are are commercially available and validated for flow cytometry applications by the suppliers

Eukaryotic cell lines

Policy information about cell lines and Sex and Gender in Research

Cell line source(s)	OM-10.1 cell line. Source: National Institute for Biological Standards and Control (NIBSC)
Authentication	OM-10.1 cell line was authenticated by the provider (NIBSC)
Mycoplasma contamination	All cell lines were tested negative for mycoplasma
Commonly misidentified lines (See ICLAC register)	na

Flow Cytometry

Plots

Confirm that:

- [x] The axis labels state the marker and fluorochrome used (e.g. CD4-FITC).
- [x] The axis scales are clearly visible. Include numbers along axes only for bottom left plot of group (a 'group' is an analysis of identical markers).
- [x] All plots are contour plots with outliers or pseudocolor plots.
- [x] A numerical value for number of cells or percentage (with statistics) is provided.

Methodology

Sample preparation	All sample preparation details and methods are detailed in the method section
Instrument	BD LSR-II
Software	BD CellQuest for acquisition, FlowJo 10.0.7r2 for analysis.
Cell population abundance	The abundance of cell population post sorting, their purity and determination methods are all detailed in the text and appropritate figures and in the method section. Tissues were processed to obtain a tissue cell suspension of urethral mucosal cells as described in Ganor et al., 2019. Entire tissue cell suspensions contain around 100 million cells per individual after tissue proccessing. Magnetic selection of macrophages from tissue cell suspensions yeild around 1 million macrophages per individual. Viable macrophages corresponded to >90% (before) and >95% (after dead cell removal) of cells among the population of total tissue macrophages (CD68+) in the cell suspension.
Gating strategy	Gating strategy is cleraly detailled in the method section and in relevant figures. Dead cells were excluded gating out cells with low FSC/SSC (as validated using DRAQ7 staining); Doublets in viable cell population were excluded in an FSC-A/FSC-H dot plot. Macrophages were gated in a CD3/CD68 dot plot as CD68+CD3neg cell population. M4 macrophages are gated as S100A8+MMP7+ CD68+ cells.

- [x] Tick this box to confirm that a figure exemplifying the gating strategy is provided in the Supplementary Information.
